# Spatiotemporal prediction of soil organic carbon density in Europe (2000–2022) using earth observation and machine learning

**DOI:** 10.7717/peerj.19605

**Published:** 2025-07-14

**Authors:** Xuemeng Tian, Sytze de Bruin, Rolf Simoes, Mustafa Serkan Isik, Robert Minarik, Yu-Feng Ho, Murat Şahin, Martin Herold, Davide Consoli, Tomislav Hengl

**Affiliations:** 1 OpenGeoHub, Doorwerth, Netherlands; 2 Laboratory of Geo-Information Science and Remote Sensing, Wageningen University and Research, Wageningen, Netherlands; 3 Department of Geosciences & Engineering, Delft University of Technology, Delft, Netherlands; 4 Remote Sensing and Geoinformatics, Helmholtz GFZ German Research Centre for Geosciences, Potsdam, Germany

**Keywords:** Soil organic carbon density, Machine learning, Earth observation, Uncertainty, Spatial aggregation, Time series, Random forest, Model interpretability, Shapley value, Data transformation

## Abstract

This article describes a comprehensive framework for soil organic carbon density (SOCD, kg/m^3^) modeling and mapping, based on spatiotemporal random forest (RF) and quantile regression forests (QRF). A total of 45,616 SOCD observations and various Earth observation (EO) feature layers were used to produce 30 m SOCD maps for the EU at four-year intervals (2000–2022) and four soil depth intervals (0–20 cm, 20–50 cm, 50–100 cm, and 100–200 cm). Per-pixel 95% probability prediction intervals (PIs) and extrapolation risk probabilities are also provided. Model evaluation indicates good overall accuracy (*R*^2^ = 0.63 and CCC = 0.76 for hold-out independent tests). Prediction accuracy varies by land cover, depth interval and year of prediction with the worst accuracy for shrubland and deeper soils 100–200 cm. The PI validation confirmed effective uncertainty estimation, though with reduced accuracy for higher SOCD values. Shapley analysis identified soil depth as the most influential feature, followed by vegetation, long-term bioclimate, and topographic features. While pixel-level uncertainty is substantial, spatial aggregation reduces uncertainty by approximately 66%. Detecting SOCD changes remains challenging but offers a baseline for future improvements. Maps, based primarily on topsoil data from cropland, grassland, and woodland, are best suited for applications related to these land covers and depths. We recommend that users interpret the maps in conjunction with local knowledge and consider the accompanying uncertainty and extrapolation risk layers. All data and code are available under an open license at https://doi.org/10.5281/zenodo.13754343 and https://github.com/AI4SoilHealth/SoilHealthDataCube/.

## Introduction

Organic carbon stored in soils is of increasing interest to policy makers as a key indicator of land productivity and a potential global solution to offset greenhouse gas emissions from agriculture and combat climate change (Friedlingstein et al., 2014; Lamb et al., 2016; Jackson et al., 2017; Smith et al., 2020; Panagos et al., 2024). This storage is quantified as soil organic carbon density (SOCD, kg/m^3^), which represents the total amount of organic carbon in a given volume of soil. SOCD is sometimes referred to as “SOC stocks” (in kg/m^2^ or t/ha) when specified to a particular depth interval. It also serves as a key indicator for the Land Degradation Neutrality initiative under the United Nations Convention to Combat Desertification (Mattina et al., 2018). Given its critical role, there is a growing demand for up-to-date, dynamic, and spatially continuous information of SOCD (National Academies of Sciences, Engineering, and Medicine, 2021; De Rosa et al., 2024; Keel et al., 2021).

Recent advances in data-driven digital soil mapping (DSM), especially leveraging machine learning (ML) to handle complex non-linear relationships and the increasing availability of Earth observation (EO) data, have significantly improved SOCD modeling (Veronesi & Schillaci, 2019; Hengl & MacMillan, 2019; Wadoux, Minasny & McBratney, 2020; Tziolas et al., 2024). De Brogniez et al. (2015) generated a 250 m EU-wide SOC map as a single snapshot, while Poeplau et al. (2020c) and Chen et al. (2023) assessed topsoil carbon stocks for single time periods in Germany and France, respectively. To capture SOC dynamics, temporal variables have been added to DSM, including trends in environmental factor time series (Yang et al., 2022), decayed normalized difference vegetation index (NDVI) (Heuvelink et al., 2020), and time-series EO data (Tayebi et al., 2021). These advances have extended SOC mapping over temporal scale. For example, Szatmári, Pásztor & Heuvelink (2021) estimated SOC stock changes in Hungary between 1992 and 2010 using 100 m SOC stock maps, while the World Soils project (Van Wesemael et al., 2024) produced 100 m resolution EU SOC maps for periods after 2018. Beyond Europe, Ugbaje et al. (2024) mapped SOC stocks in Australia at 90 m resolution for 1990 and 2018, MapBiomas (2023); De Sousa et al. (2024) provided annual topsoil SOC stock predictions for Brazil at 30 m resolution from 1985 to 2021, and Venter et al. (2021) modeled SOC stock long-term averages and trends for Southern Africa spanning 1984–2019. As a critical dimension of soil, depth has recently been incorporated into DSM. Following a spatio-temporal interpolation approach for soil properties tested at a small scale (Gasch et al., 2015), Helfenstein et al. (2024a) produced detailed 25 m three dimensional space and time (3D+T) soil organic matter (SOM) maps for the Netherlands, spanning 1953–2023. With these advancements, the availability of mapping products has expanded, evolving from static spatial (2D) maps to time-series maps (2D+T) and incorporating depth (3D+T).

However, generating high-resolution maps often comes at the expense of broader spatial coverage or temporal resolution due to computational constraints, making large-scale high-resolution SOCD maps relatively uncommon. Although the Land Use and Coverage Area Frame Survey (LUCAS) soil monitoring project (Orgiazzi et al., 2018) has significantly expanded data availability, to our knowledge, no high-resolution SOCD dataset currently covers the European continent over a long period (>10 years), *i.e.* which allows for a long-term trend assessment.

In this article, we provide complete and consistent predictions of SOCD across pan-EU using a reproducible and updatable automated soil mapping framework. This is achieved by integrating more than 45,000 reference SOCD measurements—harmonized from LUCAS and national legacy soil datasets—with a diverse range of environmental features, particularly time-series EO data at 30 m resolution (Tian et al., 2024b), and applying dynamic SOCD mapping in 3D+T. In addition to detailing the production of these maps, we include a thorough validation of the model’s performance across different land covers, soil depth intervals, and years. We also use Shapley values to gain deeper insight into how environmental features influence model predictions, providing a better understanding of the model’s behavior and underlying drivers (Wadoux, Minasny & McBratney, 2020).

Our methodology follows four main steps:

1. Develop a framework for modeling SOCD that optimizes performance while efficiently using computational resources, utilizing data collected and harmonized from various sources across Europe;
2. Produce a time-series of 30 m SOCD maps for continental Europe at different depth intervals from 0 to 200 cm between the years 2000 and 2022. Additionally, generate quality indicator maps, including quantified uncertainty and extrapolation risk probability.
3. Explore the influence of environmental factors on SOCD predictions through explainable ML techniques.
4. Assess the accuracy and reliability of the models and maps across different spatial supports and assess their suitability to detect temporal changes in SOCD.

The data sets and maps produced are publicly available at https://doi.org/10.5281/zenodo.13754343. Detailed implementation of the modeling framework and analysis experiments is provided in Jupyter notebooks at https://github.com/AI4SoilHealth/SoilHealthDataCube/tree/main/SOCD_map. Portions of this text were previously published as part of a preprint Tian et al. (2024a).

**Figure 1:**
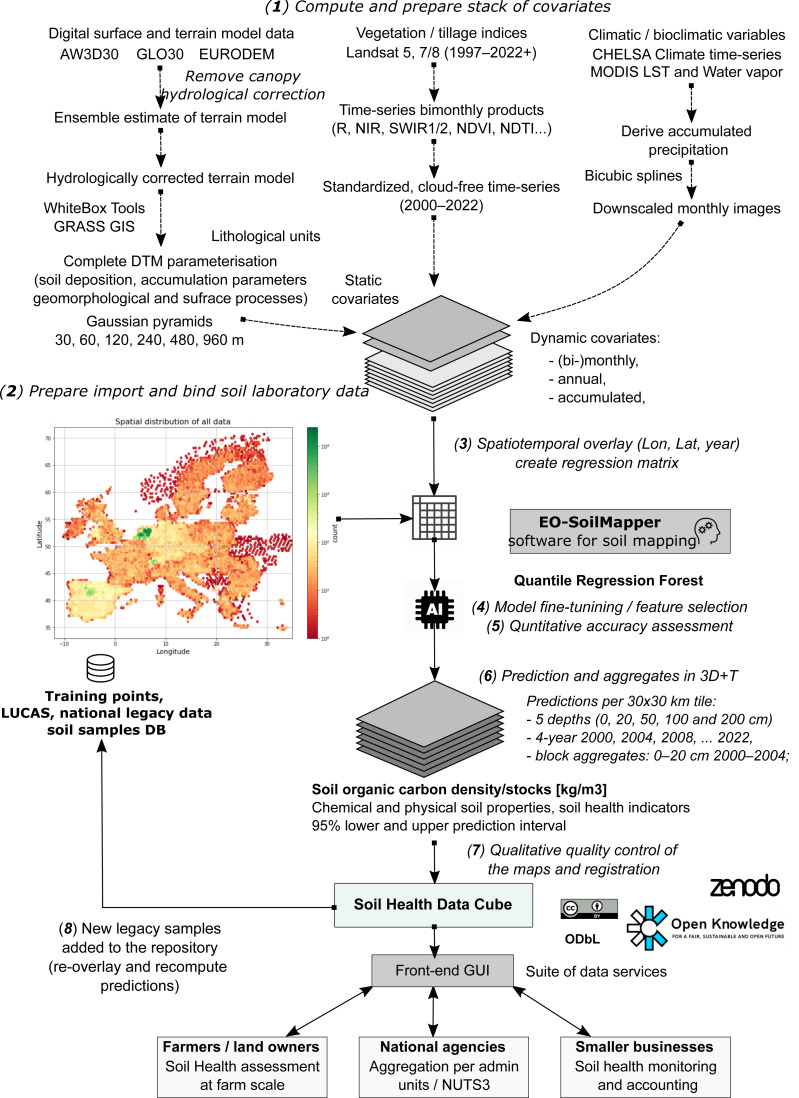
General eight-step framework for generating predictions as a part of the Soil Health Data Cube (AI4SoilHealth project). This is implemented as an automated workflow, allowing predictions to be updated and improved as new legacy soil data are harmonized and added to the training pool. Abbreviations: AW3D30, ALOS World 3D 30 m Digital Surface Model (Japan Aerospace Exploration Agency, 2021); GLO30, Copernicus GLO-30 Digital Surface Model (European Space Agency, 2024); NIR, Near Infrared; SWIR, Short-wave infrared; NDVI, Landsat Normalized Difference Vegetation Index; NDTI, Normalized Difference Tillage Index; MODIS, The NASA’s Moderate Resolution Imaging Spectroradiometer; NUTS3, EU’s small regions based on the NUTS (Nomenclature of territorial units for statistics) classification; LST, land surface temperature, MODIS.

## Material and methodology

### Spatiotemporal ML for 3D+T data

To produce consistent and seamless SOCD predictions across the EU, we developed a standardized and modular automated soil mapping framework using spatiotemporal ML and 3D+T data (Fig. 1). We adopted Random Forest (RF) as the core algorithm to predict SOCD and estimate associated uncertainties due to its proven effectiveness in SOCD mapping (Hengl et al., 2018; Wadoux, Minasny & McBratney, 2020) and its adaptability to model uncertainty, namely through the quantile regression forest (QRF) (Meinshausen, 2006; Vaysse & Lagacherie, 2017). The spatiotemporal ML framework is based on eight steps that include (Fig. 1): (1) preparing feature layers, (2) preparing, importing and binding soil laboratory (training) data, (3) spatiotemporal overlay and generation of regression matrix, (4) model training, including feature selection and parameter fine-tuning, (5) quantitative accuracy assessment through cross-validation (CV) and individual test, (6) prediction, (7) qualitative quality control through visual checks to identify artifacts, gaps, overfitting, and potential problems, and (8) map updates. This framework is most similar to the frameworks suggested by Venter et al. (2021) and Yuzugullu et al. (2024), with the difference that our framework is optimized for producing predictions in 3D+T with uncertainty mapped per pixel. This general modeling design is the basis of the Soil Health Data Cube, which is fully documented at https://shdc.ai4soilhealth.eu/.

Our models and output maps (predictions) are designed to be updatable; when reviewed by experts, spatial, temporal, or feature-space gaps can be identified, indicating where additional data and features are needed. As new data or improved features become available, the framework can be re-run to refine and update the maps. The eighth step, re-analysis, is especially dependent on receiving more European legacy soil laboratory data (training points) through emerging collaborations and initiatives, allowing us to continuously expand and update our pool of training points and gradually make more accurate predictions.

Note that detecting SOCD changes from LUCAS soil points is challenging, even though LUCAS provides repeated soil samples. Soils in general have complex and slow-changing nature (Poeplau & Don, 2013; Smith et al., 2020), and this challenge also applies to modeled SOC, even on an extended time scale such as 1953–2022, as highlighted by Helfenstein et al. (2024b). Thus, prediction uncertainties need to be reported at the pixel level, for example, by using the 95% prediction intervals (PI). This helps facilitate effective communication with end users and ensure proper application (Arrouays et al., 2020a).

We conducted an exploratory analysis to assess the potential for detecting temporal changes by evaluating whether the modeled time series exceed the PIs, providing preliminary insight into the suitability of predictions for change detection. This temporal analysis is conducted at multiple levels, including the pixel level and, in this study, small regions (*e.g.*, county) of Nomenclature of Territorial Units for Statistics (NUTS3) administrative units level through a case study, similar to the approach taken by Szatmári, Pásztor & Heuvelink (2021) and Szatmári et al. (2024) in Hungary. We believe that such multi-scale analysis is relevant for applications across varying spatial contexts (Piikki & Söderström, 2019).

In addition to PI, we calculated anomaly probability maps to indicate the probability of extrapolation (risk) in the model. These maps provide users with guidance on areas where additional caution is required. Beyond generating and assessing maps and models, we analyzed feature importance with explainable machine learning techniques to understand how environmental features influence predictions, offering transparency into the model’s behavior, and guiding future refinement.

### Point data collection and harmonization

To calculate SOCD (kg/m^3^), three key properties are required: SOC (content, g/kg), bulk density (BD, g/cm^3^), and coarse fragments (CF, %). As described in Poeplau, Vos & Don (2017), there are two paths to compute SOCD, depending on the availability of the data. The first approach uses total bulk density (BD_tot_) and coarse fragments by mass (CF_mass_), as described in Eq. (1):

(1) \begin{eqnarray*}\text{SOCD}= \frac{\text{SOC}\cdot {\text{M}}_{\text{fe}}}{{\text{V}}_{\text{tot}}} = \frac{\text{SOC}\cdot {\text{M}}_{\text{tot}}\cdot (1-0.01\cdot {\text{CF}}_{\text{mass}})}{{\text{V}}_{\text{tot}}} =\text{SOC}\cdot {\text{BD}}_{\text{tot}}\cdot (1-0.01\cdot {\text{CF}}_{\text{mass}}).\end{eqnarray*}

The second approach utilizes fine earth bulk density (BD_fe_) and coarse fragments by volume (CF_vol_), as shown in Eq. (2):

(2) \begin{eqnarray*}\text{SOCD}= \frac{\text{SOC}\cdot {\text{M}}_{\text{fe}}}{{\text{V}}_{\text{tot}}} = \frac{\text{SOC}\cdot {\text{M}}_{\text{fe}}\cdot (1-0.01\cdot {\text{CF}}_{\text{vol}})}{{\text{V}}_{\text{fe}}} =\text{SOC}\cdot {\text{BD}}_{\text{fe}}\cdot (1-0.01\cdot {\text{CF}}_{\text{vol}}).\end{eqnarray*}

Data on SOC, BD, and CF were collected from a range of regional, national, and pan-European surveys at the sample level, with each depth treated separately. The quality of these datasets varied, some datasets provided detailed information on soil depth, sampling time, sampling methods, measurement methods, and units, while others lacked such essential information. To ensure data consistency and reliability, standards were applied during data filtering and cleaning. Precise information on sampling year and geographic coordinates was required for spatiotemporal overlay with feature data to enable spatiotemporal modeling. Soil depth data was harmonized as follows: where a single value was recorded, it was directly used as the soil depth; where upper and lower depth limits were recorded, their mean value was calculated and used. The soil depth was then used as a feature in the modeling process, enabling the prediction of SOCD at various depths. Records missing any of these critical details were excluded.

For BD, we used laboratory measurements of the oven-dry mass of soil per unit volume, explicitly distinguishing between the fine earth density and the total bulk density. CF data were included only if they specified fragments larger than two mm and clearly indicated whether the measurements referred to mass or volume. SOC harmonization was guided by the LUCAS dataset, which served as the benchmark due to its high-quality data, extensive coverage, and standardized methodology across Europe. Measurements compatible with the dry combustion method, as used in the LUCAS soil survey to determine SOC, were converted using established conversion factors. For example, although the conversion factor between the Walkley-Black method and dry combustion varies slightly between studies, the differences are generally minimal, and therefore we adopted the widely recognized factor of 1.3 (Walkley & Black, 1934; Kumar, Ghotekar & Dadhwal, 2019; Shamrikova et al., 2022). Data measured using incompatible methods or those lacking methodological details were excluded. An example of an incompatible method is loss on ignition (LOI), where conversion factors vary significantly across studies, ranging from 0.3 to 1.1 (Soon & Abboud, 1991; Konen et al., 2002; Kumar, Ghotekar & Dadhwal, 2019; Hernández et al., 2023). Furthermore, point data with zero SOCD values were excluded from the study if the associated land cover data did not indicate bare earth, rock, or sand.

After cleaning and harmonizing the data, we retained records that contained either all three properties—SOC, BD_tot_, and CF_mass_—or SOC, BD_fe_, and CF_vol_. These records were used to calculate the SOCD values using Eqs. (1) and (2), as described in Table 1. In addition, we included a small *“pseudo-zero”* SOCD dataset to address gaps in the feature space caused by the absence of samples from areas with minimal or no soil. These locations are often unintentionally omitted from field sampling campaigns due to practical reasons (Barbet-Massin et al., 2012; Hengl et al., 2017). The *“pseudo-zero”* dataset was derived from land cover points identified as bare rock or shifting sand in the GLanCE project, where 20 cm resolution very high resolution (VHR) imagery was used to characterize such areas (Stanimirova et al., 2023). It was used during model calibration and training to help the model recognize covariate patterns associated with non-soil environments. Without such examples, the model could overestimate SOCD values in barren areas.

**Table 1:** Count of SOCD measurements by sources and associated information.

**Source**	**Derivation method**	**SOC method**	**Measurement count**	**Location count**	**Max depth (cm)**
LUCAS	Eq. (2)	Dry combustion	5,806	5,806	10
BZE-LW	Eq. (2)	Dry combustion	16,234	2,938	184.5
Parcelas COS	Eq. (1)	Walkley-Black	1,576	788	20
Parcelas INES	Eq. (1)	Walkley-Black	20,656	20,652	5
Infosolo	Eq. (1)	Dry combustion	252	100	90
Infosolo	Eq. (1)	Springer & Klee	106	25	179
Infosolo	Eq. (1)	Walkley-Black	266	126	140
GLanCE	Estimation	Estimation	720	720	5

**Figure 2:**
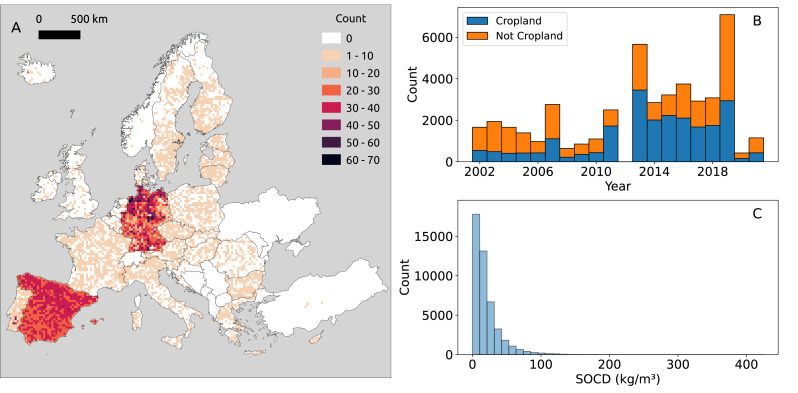
Distribution of point SOCD (kg/m^3^) data. (A) Spatial distribution of SOCD across continental Europe, with each grid cell representing an area of 25 km^2^; (B) Temporal distribution of SOCD from 2000 to 2020; (C) Histogram of SOCD values.

In total, we compiled 45,616 SOCD point data from 30,762 unique locations across Europe with valid environmental feature values (Table 1). The spatial distribution of this dataset is illustrated in Fig. 2A. The LUCAS dataset serves as the cornerstone of the SOCD dataset. Although not the largest data source, it ensures a broad spatial distribution of samples across Europe. We specifically used BD_fe_ data derived by Pacini et al. (2023) from raw LUCAS BD data, which includes corresponding CF_vol_ values, facilitating the derivation of SOCD. National datasets further enriched the SOCD data but also introduced some clustering in specific regions. Another major data source is the systematic Spanish soil survey datasets, Parcelas COS (to 30 cm depth) and Parcelas INES (top 10 cm) (Serrano et al., 2022), which provided the majority of SOCD data, primarily from topsoil. BZE-LW, the core dataset of the first German agricultural soil inventory (Poeplau et al., 2020b; Poeplau et al., 2020a), is the second largest data source, offering more diverse data across soil depths. The INFOSOLO database from Portugal (Ramos et al., 2017) also contributed valuable data. The SOCD data estimated from GLanCE datasets account for the majority of data from bare rock and shifting sands in the topsoil.

**Figure 3:**
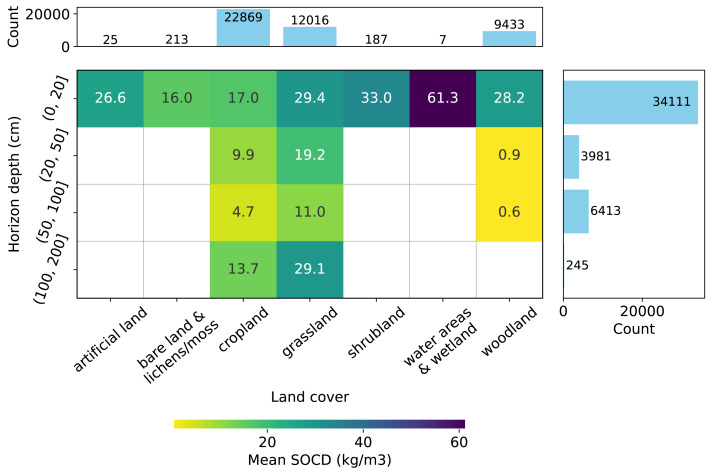
Distribution of SOCD observations (excluding *pseudo-zero* data) across land cover types and soil depth intervals. Grid cells and colors represent the mean SOCD for each land cover–depth combination.

Our compiled trainind dataset spans 2000 to 2019, with notable data concentrations in 2012 and 2018 (Fig. 2B) which are the LUCAS points. The overlap of BZE-LW and Parcelas INES datasets characterizes 2012, while 2018 corresponds to the LUCAS BD survey. The distribution of SOCD data is highly skewed, with most values concentrated around a central point and a long tail extending toward larger values (Fig. 3C). Before 2010, the majority of data points of each year were from non-cropland areas; however, after 2010, the distribution shifted, with more data points come from cropland.

Depth-wise, most samples represent the topsoil (0–20 cm), as shown in Fig. 3, while data for deeper soil layers are more limited. Data are available for all types of soil cover in the topsoil, while data for deeper depths is limited to samples from *cropland*, *grassland*, and *woodland*. Generally, the mean measured SOCD values decrease with increasing soil depth up to 100 cm. Beyond this depth, from 100 cm to 200 cm, mean SOCD values appear to increase again; however, this inconsistency is likely due to limited data availability. We can also observe that the addition of the pseudo-zero SOCD dataset significantly improves the representation of bare land and lichens or moss land covers, which account for approximately 80% of the data counts (720 out of 933). Note that the land cover information associated with the soil samples is based on the LUCAS land cover classification system, with the main classes shown in Fig. 3. For more details, see the LUCAS Technical Reference Document: C3 Classification (Land Cover & Land Use) (https://ec.europa.eu/eurostat/documents/205002/8072634/LUCAS2018-C3-Classification.pdf).

The SOCD values and the availability of the data also vary by land cover type (Fig. 3). The SOCD data from water areas and wetlands seem to exhibit the highest mean values, while the samples from bare land and lichens or moss show the lowest values. Cropland samples form the majority of the dataset, followed by grassland and woodland, while samples from other land cover types are quite limited. The land cover classifications were derived from the original data and standardized to align with the LUCAS land cover system. Note that 146 points lack valid land cover records and were excluded from this plot and any subsequent analyzes involving land cover strata.

All detailed data harmonization procedures are outlined in the Soil Health Data Cube manual (https://shdc.ai4soilhealth.eu/), which comes with the production and analysis code.

### Predictive features

A data-driven approach was used for feature selection. A wide range of features (also referred to as *“predictors”* or *“covariates”*) was included to represent various environmental factors such as vegetation, climatology, topography, human activity, and water content, *etc*. This redundancy resulted in an initial feature pool of 582 variables. Some features provided overlapping information; for example, Landsat-derived bare soil fractions (BSF) from time-series analysis may overlap with bare soil (BS) fractions derived by Sun et al. (2023) using spectral unmixing.

For time-series data with missing values for recent years (*e.g.*, 2020 and later) or earlier years (*e.g.*, before 2001), we substituted the most recent available data from surrounding years. For example, CHELSA climate time-series data ends in 2019, so data for 2020, 2021, and 2022 were copied from 2019. All data layers were resampled to a 30 m resolution using cubic splines (as implemented in GDAL) where necessary, ensuring consistency in spatial extent, resolution, and coordinate reference system. The following paragraphs detail the preparation of feature groups and the feature selection method.

The topographic variables were derived from the Ensemble Global Digital Terrain Model (EDTM) at 30 m spatial resolution and based on AW3D30, GLO30 and continental DTMs (Ho, Hengl & Parente, 2023), as the topography-factor attributes for SCORPAN model (McBratney, Santos & Minasny, 2003). These include terrain parameters such as slope (in degrees), minimum and maximum curvature, hillshade, negative, and positive openness. Hydrology parameters, including specific catchment area, slope length and steepness factor, flow accumulation, and topographic wetness index, were obtained using SAGA GIS (Conrad et al., 2015). Furthermore, northerness and easterness (representing the north-facing and east-facing component of the slope, used as a proxy for sunlight exposure), and geomorphon classes were generated using GRASS GIS (GRASS Development Team, 2023) to capture the landscape orientation and geomorphological features, which play an important role in the pedogenetic process (Sena et al., 2020). The EDTM was resampled from 30 m to 60 m, 120 m, 240 m, 480 m, and 960 m and the derivation of parameters is repeated to produce a Gaussian pyramid (Behrens et al., 2018). Coarse-resolution layers (240 m, 480 m, and 960 m) derive terrain and hydrology parameters on a continental scale. Fine-resolution layers (30 m, 60 m and 120 m) are first divided into tiles and padded with 3,400 pixels for geomorphon classes and hydrology parameters, whereas 100 pixels for the rest. The tiles are mosaicked and then reprojected to EPSG:3035 and finally cropped to the same spatial extent as the other layers.

The climate data used in this study include precipitation, daytime and nighttime land surface temperatures (LST), water vapor, and long-term BIOCLIM variables (Karger et al., 2017). The precipitation data consist of cumulative monthly and annual cumulative sums, as well as long term average of monthly precipitation, all derived from the CHELSA daily precipitation product (Karger et al., 2021). Temperature data covers time series for monthly and annual median values, 5th and 95th percentiles, and standard deviations of daytime and nighttime LST, sourced from the MODIS LST product (Wan, 2006). Water vapor data includes monthly mean and standard deviation, as well as annual median, 25th, and 75th percentiles and standard deviation, derived from the MODIS water vapor product (Lyapustin & Wang, 2018), as processed by Parente, Simoes & Hengl (2023). Long-term climate characteristics, such as annual trends (*e.g.*, mean annual temperature, annual precipitation), seasonality (*e.g.*, annual temperature and precipitation ranges) and extreme or limiting environmental factors (*e.g.*, temperatures of the coldest and warmest months and precipitation during the wettest and driest quarters), are represented by CHELSA BIOCLIM variables (Karger et al., 2017; Brun et al., 2022).

We produced a gap-free lithology map for Europe by imputing missing information from the EGDI/OneGeology (https://maps.europe-geology.eu/#baslay=baseMapGEUS&extent=295409.9588900306,1155970,8209570.04110997,5309410&layers=onegeoeuro_surface_lithology) surface geology map using a RF classifier trained on topographic variables and soil region maps (BGR, Bundesanstalt für Geowissenschaften und Rohstoffe, 2005). For Türkiye, lithology was digitized and harmonized using the geological map from MTA (https://atag.itu.edu.tr/v4).

To consider the influence of human activities on SOCD, several variables were included to capture the extent and intensity of human pressure, including Human Footprint Index, nightlight data and cropland extent. Human Footprint Index, developed by Mu et al. (2022) combines data on land cover, accessibility, nightlights, and population density into a scoring scheme to estimate human pressure. Annual visible nightlight data (V2) from NASA/NOAA’s Visible Infrared Imaging Radiometer Suite for the period 2012–2019 (Elvidge et al., 2021), extrapolated for 2000–2011 using logistic regression by Hengl (2023), were included as a proxy for human activity and land-use intensity, which could potentially influence land productivity there influence SOCD (Hackländer et al., 2024). The global maps for cropland extent developed by Potapov et al. (2022) are adopted to indicate the possible human impact on the soil related to agricultural practices, such as crop residues and soil disturbance patterns. This cropland dataset includes annual and perennial herbaceous crops, excluding woody crops and permanent pastures.

Land cover dynamics, particularly those involving vegetation and soil components, is essential to understand SOCD dynamics. To quantify land cover changes over time, we used two datasets derived from distinct methods, both providing continuous numerical representations of land cover rather than hard classes. The first dataset represents the annual mean, maximum and standard deviation of photosynthetic vegetation, non-photosynthetic vegetation, and bare soil (BS) fractions. These metrics were derived from the monthly Vegetation and Soil Fractions (2001–2022) product, generated by spectral unmixing of reflectance images (Sun et al., 2023). The second data set is the Plant Functional Types (PFT) Maps (1992–2020) (Harper et al., 2023), which were produced using the European Space Agency’s Climate Change Initiative (CCI) land cover data product. This dataset, created using a cross-walk table and auxiliary data, provides annual compositions of 14 PFTs at 300 m resolution, capturing intra-class spatial variability for detailed surface process representation.

To better represent soil surface structure, we used the normalized mean radar backscatter coefficients from the Sentinel-1 Global Backscatter Model (S1GBM), which was produced using synthetic aperture radar (SAR) images covering 2016–2017 period and was corrected for incidence angle variations to achieve consistency across the globe (Bauer-Marschallinger et al., 2021). The mosaics of C-band dual polarization backscatters (VV/VH) were downsampled from 10 m to 30 m spatial resolution to comply with high resolution features. The use of SAR images has been shown to be effective in mapping various soil compositions, such as soil moisture (Celik et al., 2022), soil type (Deodoro et al., 2023), and soil organic carbon (Nguyen et al., 2022).

Although most other features are initially available at coarser resolutions and later resampled to finer scales, we used a 30 m Landsat-based spectral indices data cube as the foundation for our 30 m spatial resolution SOCD mapping. This data cube, developed by Tian et al. (2024b) from Landsat ARD V2 data (Potapov et al., 2020) using the method of Consoli et al. (2024), includes both surface reflectance bands (*e.g.*, red, blue) and derived spectral indices such as the NDVI, NDTI, BSF, *etc*. These indices capture various environmental factors, including vegetation, soil, crops, and water. In addition to its extensive range of indices, the data cube is available at multiple temporal resolutions—bimonthly, annual, and long-term (2000–2022)—providing temporal characteristics at various levels.

### Model calibration

#### Spatiotemporal overlay

The spatiotemporal overlay links point observations with corresponding environmental feature values based on location and year, generating a regression matrix for model calibration and evaluation. Environmental features are broadly divided into two groups: dynamic features and static features. Dynamic features represent environmental processes that vary spatially and temporally, with values changing over time. In contrast, static features are location-specific and remain constant over time for a given location. These typically include relatively stable environmental factors (*e.g.*, lithology or topographic features) or aggregated representations of dynamic features over a long period (*e.g.*, the 50th percentile of NDVI from 2000 to 2022).

For dynamic features, the sampling year of each point observation was used to align it with the corresponding feature values. For features with finer temporal resolutions, such as bimonthly (one value per two months) or monthly (one value per month), which vary within a year, all temporal values available for the feature within the observation year are overlaid with the point observations. The overlay operation was performed using the SpaceTimeOverlay function from the scikit-map package (Consoli et al., 2024).

#### Data split, usage and transformation

The overlaid dataset was then divided into three subsets: a calibration set, a training set, and a test set. This split is done using a stratified approach to ensure representativeness across different conditions, with strata defined by combinations of depth intervals and land cover types, as shown in Fig. 3. Pseudo-zero points were excluded from the hold-out test set, as they are not based on actual measurements and the cluster of data at origin could cause potential distortion of performance metrics. Approximately 10% of the data was selected as the calibration set, which was used to establish the model structure, including feature selection and hyperparameter tuning. This separation helps improve model generalizability and reduce overfitting. The training set was used for model training and CV, while the independent test set was excluded from both calibration and training to provide an unbiased validation. The final subsets included 4,985 point measurements for model calibration, 38,348 for training, and 2,283 for testing.

The distribution of SOCD data is highly skewed, making data transformation beneficial for effectively applying geostatistical methods (Osborne, 2010; Martin et al., 2014). An experiment detailed in the supplementary notebook 005_transformation_comparison (https://github.com/AI4SoilHealth/SoilHealthDataCube/blob/main/SOCD_map/005_transformation_comparison.ipynb) shows that transforming SOCD data also enhances the performance of RF models, despite their non-parametric nature. Therefore, the RF model in this study was applied to log-transformed SOCD data, with predictions back-transformed before being presented to users as maps.

#### Extended random forest

The RF was selected for this study to model and map SOCD due to its demonstrated effectiveness in handling large datasets and capturing complex non-linear relationships (Hengl et al., 2018; Wadoux, Minasny & McBratney, 2020). Its extension, QRF (Meinshausen, 2006), quantifies uncertainty by providing estimates of conditional quantiles from the full distribution of predictions across all trees in the forest, rather than focusing solely on the conditional mean (Vaysse & Lagacherie, 2017; Szatmári & Pásztor, 2019; Schmidinger & Heuvelink, 2023).

In this study, we extended the RF implementation to run RF and QRF models simultaneously in a single execution. Specifically, we modified the RandomForestRegressor function to store the full output distribution from each tree in the forest. This modification allows us to compute both the conditional mean, consistent with standard RF predictions, and specific quantiles (*e.g.*, P0.025 and P0.975) for QRF functionality. This integrated approach enhances the efficiency of the modeling and mapping process, allowing both predictions and PIs to be obtained simultaneously, reducing computational overhead. The resulting implementation is available as the trees_rf function in the Python library scikit-map (Consoli et al., 2024).

#### Feature selection

The repeated subsampling-based cumulative feature importance (RSCFI) method was developed to select the most relevant features from the candidate feature pool. Similar to recursive feature elimination with cross validation (RFECV)—a standard approach in digital soil mapping (Wadoux, Minasny & McBratney, 2020)—RSCFI recursively eliminates features based on CV results. However, unlike RFECV, which removes a fixed number of features at each step, RSCFI removes features with cumulative feature importance (CFI) values below a predefined threshold at each iteration. The RSCFI is designed to balance model performance and computational efficiency. When many features have low CFI values, RSCFI efficiently removes most irrelevant features. Conversely, if features have comparable CFI values, RSCFI removes a stable number of features recursively, requiring a similar amount of time as RFECV. Both methods were tested on the calibration dataset, and the method yielding optimal performance across metrics—including the coefficient of determination (R^2^), concordance correlation coefficient (CCC; Lawrence & Lin, 1989), mean absolute error (MAE), and median absolute error (MedAE)—was selected for feature selection.

#### Hyper-parameter fine tuning

With preprocessed data and selected features, hyperparameter fine-tuning was performed using 5-fold CV with the HalvingRandomSearchCV function (Pedregosa et al., 2011), optimizing for CCC as the criterion. The initial hyperparameter space is detailed in Table 2. The number of trees (n_estimators) was set to 120, following the recommendation of Oshiro, Perez & Baranauskas (2012), to balance model performance, computational efficiency, and memory usage. This choice is also supported by our experimental results, which show no significant performance improvement when the number of trees exceeds 120 (see supplementary notebook 007_explore_hyper.parameter (https://github.com/AI4SoilHealth/SoilHealthDataCube/blob/main/SOCD_map/007_explore_hyper.parameter.ipynb)).

**Table 2:** Hyperparameter space for the RF regression model.

**Hyperparameter**	**Feature space**
n_estimators	{120}
criterion	{squared_error, absolute_error, Poisson, friedman_mse}
max_depth	{10, 20, 30}
max_features	{0.3, 0.5, 0.7, log2, sqrt}
min_samples_split	{2, 5, 10}
min_samples_leaf	{1, 2, 4}

The hyperparameter space defines key settings that influence the model’s performance and complexity. The number of trees (n_estimators) determines the size of the ensemble. The criterion parameter specifies the function used to evaluate the quality of the splits, such as *squared error* or *absolute error*. The maximum depth of a tree (max_depth) controls its complexity to prevent overfitting, while the max_features parameter determines the number of features considered for the best split, expressed as a fraction or function. The min_samples_split parameter sets the minimum number of samples required to split an internal node, influencing the model’s simplicity. Similarly, min_samples_leaf specifies the minimum number of samples needed at a leaf node, restricting the size of terminal nodes and helping to avoid overfitting.

### Model evaluation

Once the model structure was established with the calibration set, the RF model was trained and evaluated using the training and test datasets. Predictions were generated by averaging the output distribution of all trees. Model performance for SOCD predictions was assessed through three components: (1) 5-fold inverse sampling-intensity weighted cross-validation (ISIW-CV) on the training dataset, where data were randomly partitioned into folds and lower weights were assigned to more spatially clustered observations to address spatial sampling bias (De Bruin et al., 2022); (2) leave-one-year-out cross-validation (LOYO-CV) on the training dataset to assess the model’s temporal transferability, in which data from an entire year were held out as the test set in each iteration (Huang et al., 2024); and (3) independent testing on a withheld test set, stratified by depth and land cover type. In both (1) and (2), CV was performed on depth-specific observations. Performance metrics—including MAE, bias, R^2^, and CCC—were calculated for all assessment components using only real measurements. Pseudo-zero points were included solely during model training and excluded from metrics calculation, as their inclusion could disproportionately affect the metrics due to their clustering near the origin. For the independent test, performance metrics were also reported for different years, and combinations of land cover and soil depth strata.

PIs are derived by calculating the conditional quantiles of SOCDs, specifically the 95% PI, with lower and upper bounds set at the 2.5th and 97.5th percentiles (P0.025 and P0.975) (Meinshausen, 2006). To evaluate the reliability of the PIs, several validation metrics are calculated on the test set, as recommended by Schmidinger & Heuvelink (2023), Goovaerts (2001), and Vaysse & Lagacherie (2017):

• **Prediction interval coverage probability (PICP):** Assesses accuracy by calculating the fraction of true values falling within the prediction intervals.
• **Prediction interval width (PIW):** Evaluates sharpness by measuring the width required to capture the predictions accurately.
• **Quantile coverage probability (QCP):** Measures the asymmetry in the uncertainty coverage. It has the same underlying logic as PICP, but it evaluates single-quantile predictions, thereby highlighting the symmetry of the PIs.
• **Accuracy plot:** Evaluate the model’s ability to predict local uncertainty by means of a scatter plot of the estimated *versus* observed fractions for different PI values.

Among these, the PIW and PICP are also reported for different combinations of land cover and soil depth strata.

### Extrapolation risk probability from anomaly scores

Extrapolation decreases ML performance, but is unavoidable in large-scale spatial mapping, making communication of such uncertainties essential. Various methods exist to identify predictions made in dissimilar feature spaces, such as the area of applicability (Meyer & Pebesma, 2021), Isolation Forest (IF) (Liu, Ting & Zhou, 2008), and homosoils (Nenkam et al., 2022). Considering the computational demands and the extensive mapping scope of this study, in the end, we decided to choose IF for its efficiency and applicability for non-normal multivariate datasets (Liu, Ting & Zhou, 2008). In most of applications of IF, it is used in fact to determine probability of inclusion using occurrence only records (Song & Estes, 2023).

The IF identifies regions outside the training data range by randomly partitioning the data and isolating samples. The ensemble.IsolationForest implementation from scikit-learn (Pedregosa et al., 2011) computes an anomaly score by averaging the path lengths over a forest of (*i.e.,* ensemble of random trees), with shorter path lengths to indicate higher anomaly. Samples located in low-density or unfamiliar regions of the feature space typically require fewer splits and thus have shorter average path lengths. The average number of splits—*i.e.,* the average path length across the dataset—is used as a threshold to distinguish between regions that lie within or outside the training feature space (Liu, Ting & Zhou, 2008). To communicate extrapolation risk, we transformed the anomaly scores produced by IsolationForest into a normalized scale from 0 to 1, where higher values represent greater extrapolation risk for a given sample or pixel. The threshold for distinguishing in-sample *vs.* out-of-sample regions was also rescaled to match this normalized scale, ensuring consistency with the extrapolation risk probability maps provided to end-users.

### Map prediction

**Figure 4:**
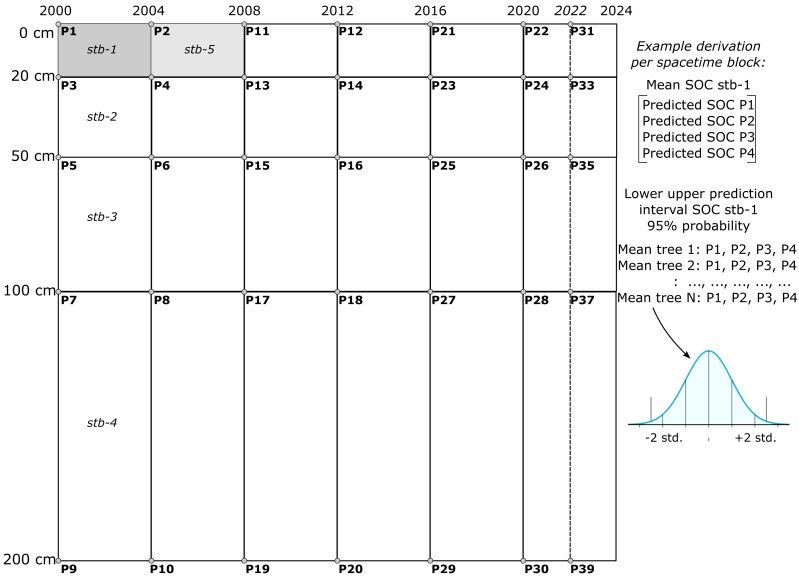
Illustration of SOCD prediction and PI generated in spacetime blocks across different temporal and depth intervals (3D+T) during map production. One spacetime block (*e.g.* stb1) is produced by averaging four predictions P1–4, while the uncertainty is derived by estimating distribution for *N* means per tree. This aggregation was chosen to reduce computational complexity and to reduce inter-annual variability effects of weather on mapping SOCD.

The final SOCD model was trained using all available SOCD data for the mapping purpose. The map production was performed using the Python library scikit-map (Consoli et al., 2024). To balance temporal resolution and computational resources, a four-year interval was chosen for this analysis, with potential extensions to finer temporal resolutions, such as annual predictions. This final single model is applied to generate SOCD predictions were generated for multiple depths (0 cm, 20 cm, 50 cm, 100 cm, and 200 cm) across different years (2000, 2004, 2008, 2012, 2016, 2020, and 2022). A spacetime block method was developed to adjust the output value of each RF tree considering a particular depth interval and a specific time interval by averaging predictions from consecutive depths and years to create ensembles of averaged tree outputs (see Fig. 4). This approach provided mean predictions and uncertainty estimates *via* PIs for four depth intervals (0–20 cm, 20–50 cm, 50–100 cm, and 100–200 cm) and six time periods (2000–2004, 2004–2008, 2008–2012, 2012–2016, 2016–2020, and 2020–2022). The standard deviation of the mean decreases by about $1/\sqrt{N}$ where *N* is the number of input prediction spacetime locations (*e.g.*, 4).

The purpose of the aggregation over *spacetime block* is to create block predictions over specific time periods and standard depth intervals, ensuring they are both representative and practical for end users. Aggregating predictions over fixed space–time intervals helps mitigate significant inter-annual variability in high-resolution covariates, such as Landsat indices, which are influenced by factors like rainfall variability, forest fires, and floods. Such variability can result in oscillating predictions that are difficult to interpret and beyond the scope and budget of this project. Aggregation reduces these fluctuations, yielding smoother distributions and more stable predictions. Furthermore, most users of SOCD data focus on SOC stocks for standard depth intervals, such as 0–20 cm or 0–100 cm. The block method simplifies the calculation of total SOCD stocks for these intervals. Consequently, our predictions can only be evaluated using block or composite measurements, such as mixing soil samples from 2–3 depths (*e.g.*, 0–20 cm) and repeating the process over 2–3 years to produce a spacetime block estimate of SOCD.

For each tile, all features selected by the model were loaded into memory for the corresponding area, year, and soil depth. The resulting tiled SOCD predictions, including uncertainties obtained by the model considering the PI, were saved as Cloud Optimized GeoTIFF files (COG). After all pan-EU tiles were produced, they were mosaicked, saved as COG, and stored in a local S3-based cloud storage, to allow visual inspection. Land masking was performed during the mosaicking process. Pan-EU land mask data used after the tiles of each product were mosaicked to create the EU-wide map.

In this study, we chose to apply the model across the entire continent, and inform users about the reliability of the predictions through guidance layers—namely, uncertainty maps, extrapolation risk maps, and performance metrics. We limit masking to buildings and permanent water bodies—areas where SOCD has no practical relevance—which do not directly correspond to the LUCAS land cover classes used in model performance evaluation. Land masks—developed based on areas of agreement across datasets to exclude permanent water bodies and built-up areas—were applied (Tian et al., 2023; DLR Geoservice of the Earth Observation Center, 2019). Note that we mask only buildings, so most urban areas, parks, and smaller farms are still included in the maps. Compared to masking land cover types with weaker performance, this approach minimizes the risk of excluding potentially valuable information. It allows results to be interpreted in the context of local knowledge, with the final decision left to the users. This rationale also applies to soil depth, for which we provide complete predictions down to 200 cm, accompanied by corresponding uncertainty information.

Extrapolation risk probability maps are provided alongside predictions, with a recommended threshold to mask areas with high extrapolation risks in the SOCD maps. Users can also adjust the threshold based on their specific needs or specific risk tolerance.

### Feature impact analysis with Shapley values

Shapley values, derived from cooperative game theory, are used in this study to interpret RF model predictions. This model-agnostic approach quantifies the contribution of each feature to the predictions, captures local variations and the importance of global features, and provides intuitive visualizations (Shapley, 1997; Padarian, McBratney & Minasny, 2020; Wadoux & Molnar, 2022; Wadoux, Saby & Martin, 2023). Using the shap package (https://shap.readthedocs.io/en/latest/) in Python, we calculated Shapley values on the test set, generating: (1) a global summary of average absolute Shapley values for general feature importance; (2) Partial Dependence Plots (PDPs) for the top 10 features; and (3) contribution plots for two locations, illustrating how features influence specific predictions. The Shapley values of the dynamic and static features are compared and analyzed, providing insight into their relative contributions and temporal dynamics in the model. It is important to note that the computation of Shapley values is intensive; therefore, it was performed only on the test dataset. Although this method provides valuable insights into the model decision-making process, its high computational cost makes it challenging to scale to large datasets.

### Quantify uncertainty of spatial aggregates

SOCD prediction uncertainty was examined over time to assess change detectability by comparing PIs with predicted changes. This analysis was conducted at two spatial supports: the pixel level and the NUTS3 regional level. At the pixel level, two individual pixels from different land covers were analyzed, while at the NUTS3 level, two regions—Unterallgäu and the area of Mindelheim Stadt within it, located in Bavaria, Germany—were merged and analyzed as a single area of interest (AOI) for demonstration purposes. The spatially aggregated SOCD in the AOI was calculated as the average predicted SOCD within the region. To quantify uncertainty in the AOI’s spatial aggregates, a method commonly applied in spatial environmental modeling (Araza et al., 2022; Wadoux & Heuvelink, 2023) was used, which accounts for the spatial autocorrelation of map errors within the aggregate:

(3) \begin{eqnarray*}{\text{sd}}_{MB}=\sqrt{ \frac{1}{{|}B{{|}}^{2}} \int \nolimits _{s\in B}\int \nolimits _{u\in B}\sigma (s)\cdot \sigma (u)\cdot \rho ({|}s-u{|})\,ds\,du}.\end{eqnarray*}

The integral in Eq. (3) was evaluated using a 500 m grid, larger than the pixel size, to balance computational efficiency and the ability to account for spatial autocorrelation within the AOI. The *B* in Eq. (3) represents the number of discretization points, which are randomly sampled from each discretized grid; *σ*(*s*) and *σ*(*u*) denote the standard deviations of the point support prediction errors at locations *s* and *u*. The *ρ*(|*s* − *u*|) represents the correlation function of the standardized prediction error at the separation distance between *s* and *u*, derived from the variogram *γ*(*h*) using Eq. (4) (Webster & Oliver, 2007, Section 4.1).

The variogram was calculated from the standardized residuals. The residuals, calculated as the differences between the map predictions and the SOCD measurements available in Germany, are standardized by dividing by the standard deviation of the map prediction error. This standardization is necessary to achieve homoscedasticity of the residuals. The standard deviation was approximated by dividing the 95% prediction interval width (P0.975–P0.025) by four, assuming a normal distribution of SOCD predictions. However, this approximation caused the variogram of standardized residuals to deviate from the expected unit sill. To address this, we applied a correction factor (the square root of the initial sill) to adjust the proxy standard deviation and ensures a unit sill upon re-standardization.

(4) \begin{eqnarray*}\rho (h)= \frac{\text{sill}-\gamma (h)}{\text{sill}} .\end{eqnarray*}

Furthermore, we calculated the average and uncertainty of this AOI using design-based (DB) estimators from the available measurement samples and compared it to the AOI mean and uncertainty quantified from the map predictions; see *e.g.,* Emick et al. (2023). The standard deviation of the mean over the NUTS3 units was calculated using Eq. (5), where *n* represents the sample size, *y*_*i*_ denotes SOCD in an individual sample within the collection and ${\bar {y}}_{DB}$ is the mean of the sampled SOCD in the AOI:

(5) \begin{eqnarray*}{\text{sd}}_{DB}=\sqrt{ \frac{1}{n(n-1)} \sum _{i\in n}{ \left( {y}_{i}-{\bar {y}}_{DB} \right) }^{2}}.\end{eqnarray*}

## Results

### Feature impact analysis

**Figure 5:**
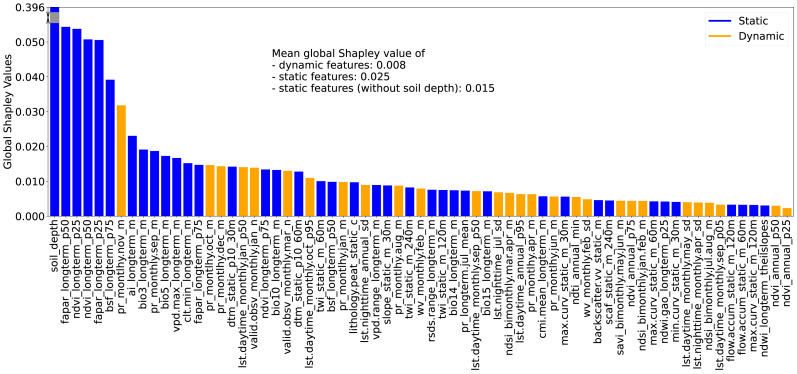
Global Shapley values for each feature, representing the feature’s contribution to SOCD prediction at each location. The global Shapley value of soil_depth is substantially higher than those of other features; therefore, it has been partially rescaled (or ‘squeezed’) into a gray scale in the plot for visual clarity. Feature names are displayed on the *x*-axis and consist of three fields: the first indicates the feature variable, the second specifies the temporal support, and the third denotes the variable type (*e.g.*, mean, standard deviation). For topographic features, a fourth field is included to indicate the spatial resolution.

The RSCFI achieves comparable model performance to RFECV while significantly reducing processing time (see supplementary notebook 006_feature.selection_rfecv.rscfi (https://github.com/AI4SoilHealth/SoilHealthDataCube/blob/main/SOCD_map/006_feature.selection_rfecv.rscfi.ipynb)). Applied to the calibration dataset using a naive RF model without parameter fine-tuning, RSCFI reduced the initial 582 features to 67, as shown in Fig. 5. The selected 67 feature set includes 33 climate features, 18 Landsat index features, and 13 topographic features. The remaining three are soil depth, VV radar backscatter, and the probability of peat lithology class (class code: 78). Among the climate features, 11 are long-term CHELSA BIOBLIM features, nine are precipitation-related, nine are LST features, two represent water vapor, and two indicate cloud coverage. Notably, no features related to human activities or land cover were selected. Static features were more likely to be kept, with 276 static and 306 dynamic variables in the original pool, compared to 39 static and 28 dynamic variables in the selected set.

Global feature importance was evaluated using the average absolute Shapley values for each feature across all predictions, with larger values indicating a stronger impact on model predictions. For simplicity and clarity, these are referred to as global Shapley values. Fig. 5 presents the global Shapley values for the 67 selected features. The soil_depth feature has the highest global Shapley value (0.396), and truncated in the figure for clarity. Static features not only outnumber dynamic features but also exhibit higher average global Shapley values (0.025), approximately three times that of dynamic features (0.008). Even when soil_depth is excluded, static features maintain a higher mean global Shapley value (0.015) compared to dynamic features.

**Figure 6:**
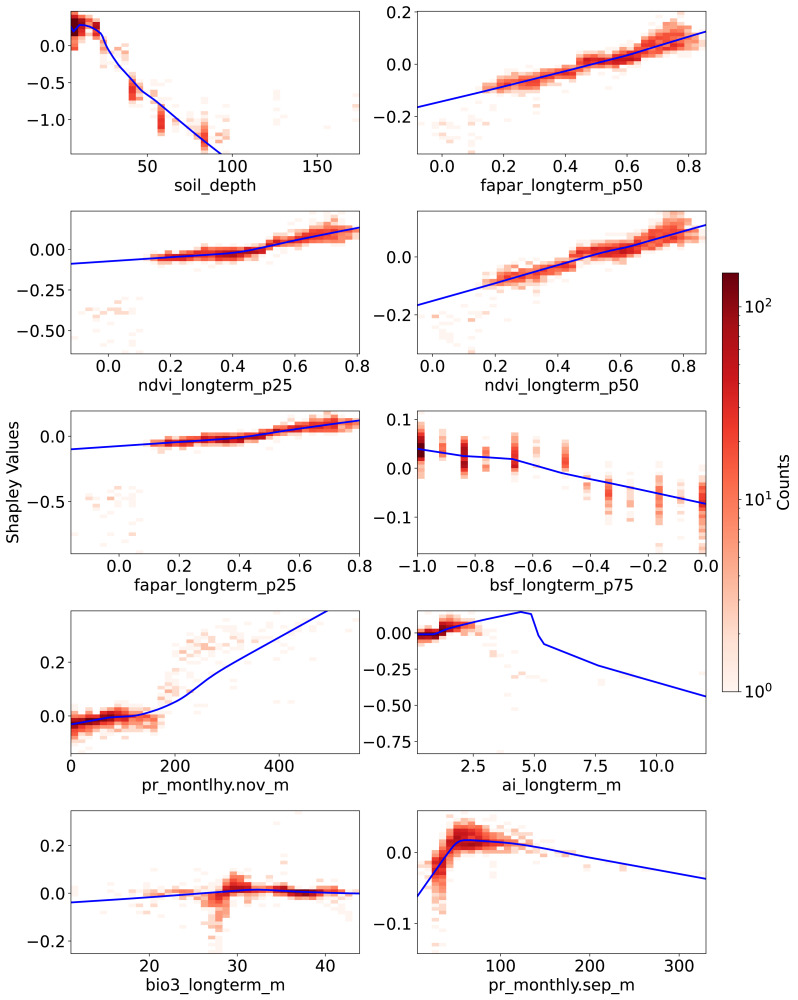
Partial dependence plots (PDPs) showing the relationship between feature values and Shapley values for the top 10 features. These plots represent the relative contribution of each feature to SOCD predictions in the test dataset. The *x*-axis indicates feature values, while the *y*-axis represents Shapley values for the respective feature. The blue line illustrates a smoothed curve fitted to the Shapley values for visualization purposes.

Figure 6 presents the PDPs of Shapley values with respect to feature values for the 10 most important features, as identified by global Shapley values. The sign of a Shapley value reflects the direction of a feature’s influence: positive values indicate that the feature increases the prediction relative to the average, while negative values suggest the opposite. For soil_depth, small values correspond to moderately high Shapley values. As soil_depth increases, Shapley values decrease, turning negative at depths greater than 20 cm. The negative influence of larger soil_depth values is particularly pronounced.

Vegetation-related features consistently exhibit a positive correlation with their corresponding Shapley values. Compared to the p25 features—ndvi_longterm_p25 (25th percentile of long-term NDVI values from 2000 to 2022) and fapar_longterm_p25—the corresponding p50 features display a more linear slope. However, data points where these four vegetation features fall below 0.2 are not well captured by the fitted PDP curves; they correspond to negative Shapley values and do not show an obvious trend. In contrast, bsf_longterm_p75 (75th percentile of long-term bare soil fraction values from 2000 to 2022), where higher values indicate barer soil surfaces, exhibits a clear negative trend in its PDP.

The last four features among the 10 most important are related to climate, three of which are associated with precipitation. For pr_monthly.nov_m (precipitation amount in November, kg m^−2^ month^−1^), Shapley values remain close to zero when feature values are below 200 but increase steeply beyond this threshold. Another precipitation feature, pr_monthly.sep_m (precipitation in September), shows a different pattern: Shapley values increase with feature values up to 100, after which they begin to decline.

The ai_longterm_mean (aridity index, defined as the ratio of mean annual precipitation to mean annual potential evapotranspiration) displays an almost linear positive trend in its PDP when values are below three, suggesting that less arid conditions correspond to higher predicted SOCD. However, when ai_longterm_mean exceeds three, the slope reverses, indicating a decreasing effect on SOCD predictions.

The temperature feature bio3_longterm_mean refers to isothermality, which is the ratio of diurnal variation to annual variation in temperatures. Its Shapley values exhibit less variation than the others. When feature value is around 30, there seems to be a clearer pattern that: when the feature values are below about 30, Shapley values tend to be negative, indicating an decreasing effect on SOCD predictions. When it is higher than about 30, the Shaply values tend to positive. But in general, The Shapley values oscillate around zero.

**Figure 7:**
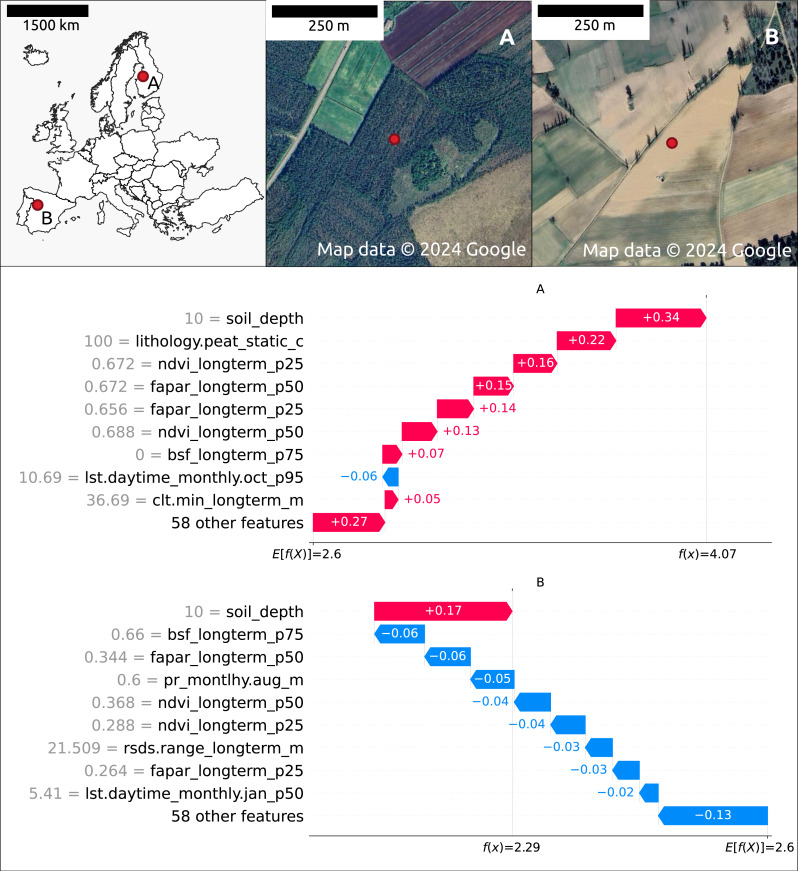
Contribution of the top 10 features to SOCD prediction at two spatial locations: (A) forested area in Finland and (B) a an agricultural area in Spain. Red indicates a positive contribution to the SOCD prediction, while blue indicates a negative contribution. Feature names and values are displayed on the *y*-axis for each location’s prediction. The decision track of SOCD predictions is shown on the *x*-axis, illustrating the progression from the starting point (average SOCD value in the test set) to the final predicted value. Satellite images from ^©^ Google Maps (2024, CNES/Airbus, Maxar Technologies), available through https://www.google.com/maps/, last accessed: 22 August 2024.

Figure 7 illustrates the feature contributions to the SOCD predictions at two distinct locations: (A) a forested area in Finland and (B) an agricultural area in Spain. The predicted SOCD values for these locations are 61.95 kg/m^3^ (4.14 on a log1p-scale) and 8.48 kg/m^3^ (2.25 on a log1p-scale), respectively, compared to observed SOCD values of 62.15 kg/m^3^ (4.15 on a log1p-scale) and 4.76 kg/m^3^ (1.75 on a log1p-scale).

The Shapley value patterns differ significantly between these two points. At point A, characterized by high vegetation coverage, a high probability of peat as the lithological class, shallow soil depth (10 cm), and relatively high clt.min_longterm_m (cloud coverage fraction, high within the test data distribution), nearly all features contribute positively to the predicted SOCD values. The only exception is lst.daytime_monthly.oct_p95 (October daytime temperature), which decreases the predicted SOCD values. In contrast, at point B, nearly all features contribute negatively to the predicted SOCD values. This point is defined by low precipitation, low vegetation coverage, and large rsds.range_longterm_m (the difference between maximum and minimum monthly surface downwelling shortwave flux in air, high in the test data distribution). The only feature contributing positively to the prediction at point B is shallow soil_depth.

The top ten features influencing SOCD predictions at these two points mostly overlap, with both sharing soil_depth and vegetation features as key features. However, different climate features are important: at point A, lst.daytime_monthly.oct_p95 and clt.min_longterm_m are significant, whereas at point B, rsds.range_longterm_m and lst.daytime_monthly.jan_p50 play a key role. Additionally, at point A, lithology.peat_static_c (the probability of lithological class peat) is also an important feature.

### SOCD model prediction accuracy

**Figure 8:**
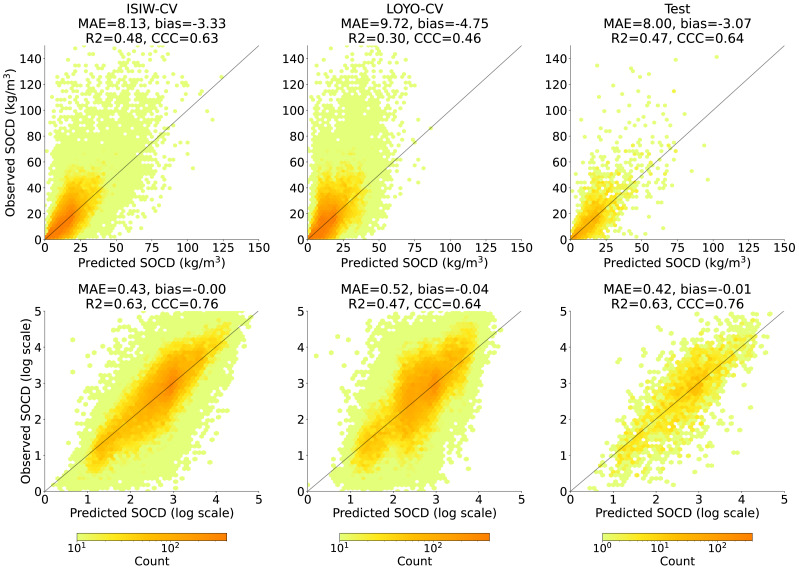
Accuracy plots in the original scale (top) generated using ISIW-CV on the training set (left), LOYO-CV on the training set (middle), and the independent test set (right), with corresponding performance metrics displayed in the titles. The bottom presents the same results in logarithmic scale. Note that SOCD values are truncated at 150 kg/m^3^ in the plots for visualization purposes; however, all performance metrics are calculated using the full range of predicted and observed SOCD values (up to 400 kg/m^3^ as shown in Fig. 2), excluding the pseudo-zero points. The same applies to the remaining figures.

**Figure 9:**
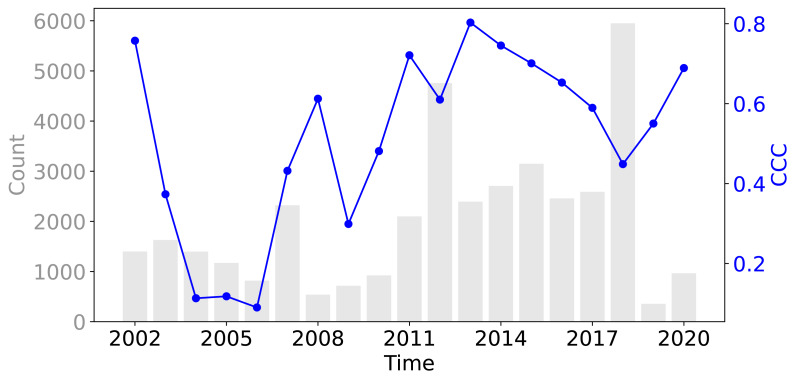
The CCC from the test dataset across different years, displayed alongside the data availability in the training dataset.

Figure 8 shows that across all three evaluation components—ISIW-CV, LOYO-CV, and the independent test: the predicted values align well with the observed values. Across the three evaluations, the independent test shows the best performance, followed by ISIW-CV and then LOYO-CV. In all validation efforts, the model exhibits a tendency to underestimate high SOCD values. In the bottom, plotted on a logarithmic scale, it is more evident that the SOCD values are also slightly overestimated for low SOCD values. Figure 9 illustrates the CCC for each year in the test dataset, highlighting substantial variation across years. No obvious correlation between CCC and data availbility is observed.

**Figure 10:**
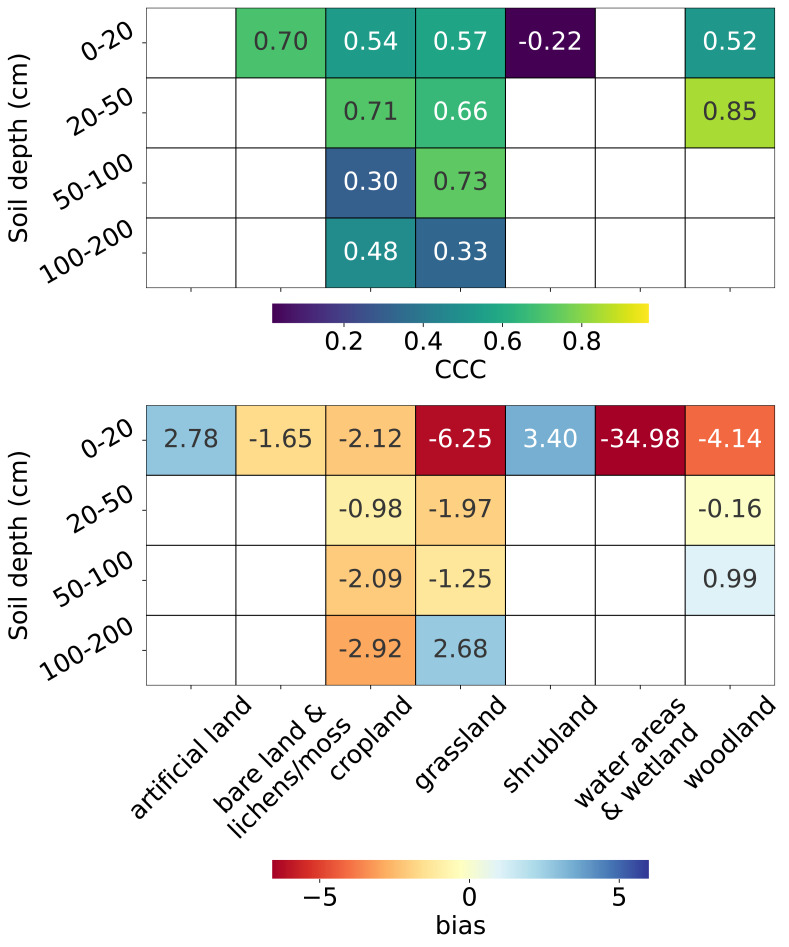
Accuracy metrics: CCC (top) and bias (bottom) for each combination of land cover and soil depth strata. Note that CCC is not shown for the 0–20 cm soil depth interval in “water areas and wetland” and *artificial land*, and for the 50–100 cm soil depth interval in *woodland* due to limited data (*n* < 5) in the test set.

From the top plot in Fig. 10, we observe that for topsoil (0–20 cm), the CCC is highest for bare land and lichens or moss (0.70), followed by grassland, cropland, and woodland (>0.5), while the lowest CCC is observed for shrubland (−0.22). Depth-wise, for land covers with soil samples deeper than 20 cm, the CCC is generally higher for the 20–50 cm depth interval than for topsoil. Only cropland and grassland have sufficient data for depths beyond 50 cm. For cropland, the CCC decreases to 0.30 in the 50–100 cm interval but increases again at 100–200 cm. For grassland, the CCC peaks in the 50–100 cm interval (0.73).

The bottom plot in Fig. 10 shows that, in general, negative biases are more prevalent than positive ones. The most pronounced underestimation occurs in water areas and wetlands (−34.98), although it is important to note that this land cover includes only seven sample points in the entire dataset (Fig. 3). The second largest underestimation is observed in the topsoil of grassland (−6.25), followed by the topsoil of woodland (−4.14). Overestimation is most notable in the topsoil of shrubland (3.40), followed by the topsoil of artificial land (2.78). For deeper soil layers, the magnitude of bias generally decreases but begins to increase again beyond 100 cm for land cover types with available samples at these depths. Plots for additional metrics across combinations of land cover and soil depth intervals are available in the supplementary notebook 008_evaluation_test.accuracy (https://github.com/AI4SoilHealth/SoilHealthDataCube/blob/main/SOCD_map/008_evaluation_test.accuracy.ipynb).

### Uncertainty estimation evaluation

**Figure 11:**
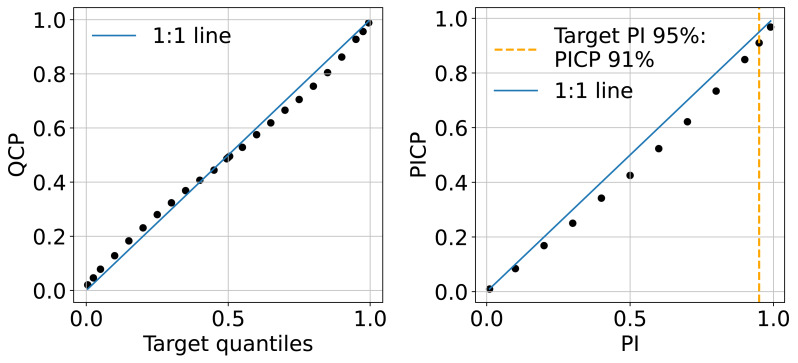
Mean QCP reliability plot (left) and mean PICP reliability plot (right) generated in the independent testing from the test set.

The QCP plots on the left of Fig. 11 indicate that the model shows slight optimism in quantile prediction. The QCP values tend to be slightly higher than the target quantiles when they are below 0.5 and slightly lower when target quantiles exceed 0.5. This pattern is also reflected in the PICP accuracy plot on the right of Fig. 11, where PICP falls below the target PIs in the middle range. Overall, the model’s uncertainty estimation aligns well with expectations. In this study, we present the 95% uncertainty map using the upper and lower limits (P0.025 and P0.975), where PICP equals 91%.

**Figure 12:**
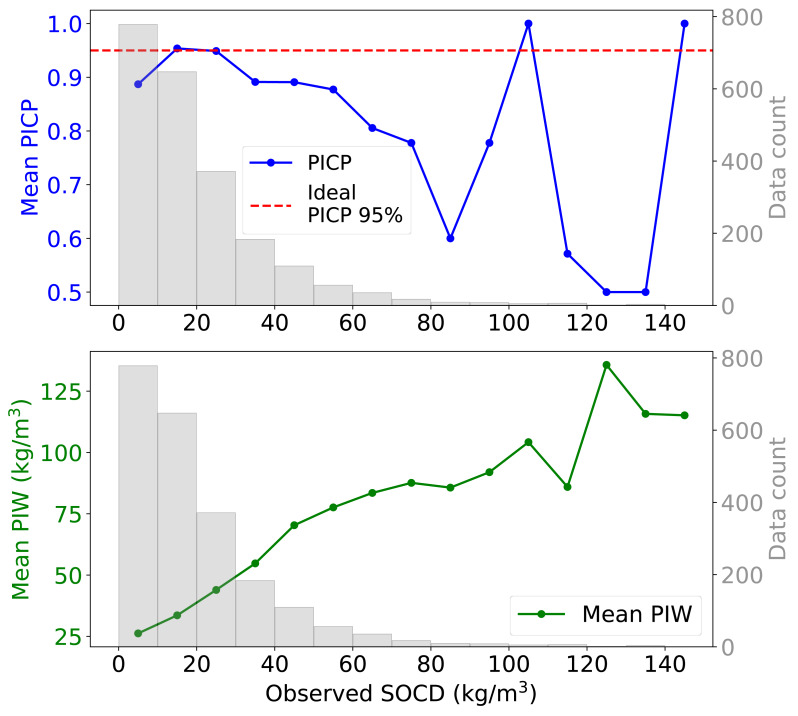
Mean PIW and PICP across different observed SOCD intervals, with the corresponding histogram.

When examining the 95% PI across varying levels of observed SOCD, the uncertainty model demonstrates inconsistent performance (Fig. 12). For SOCD values below 10 kg/m^3^, the PICP is slightly below the ideal level. Between 10 and 30 kg/m^3^, the PICP aligns well with the target level. However, as SOCD values exceed 30 kg/m^3^, the PICP generally declines with decreasing data availability. The PIW increases steadily as the availability of data decreases with increasing SOCD values. For SOCD values above 100 kg/m^3^, both PICP and PIW become unstable due to very limited data.

**Figure 13:**
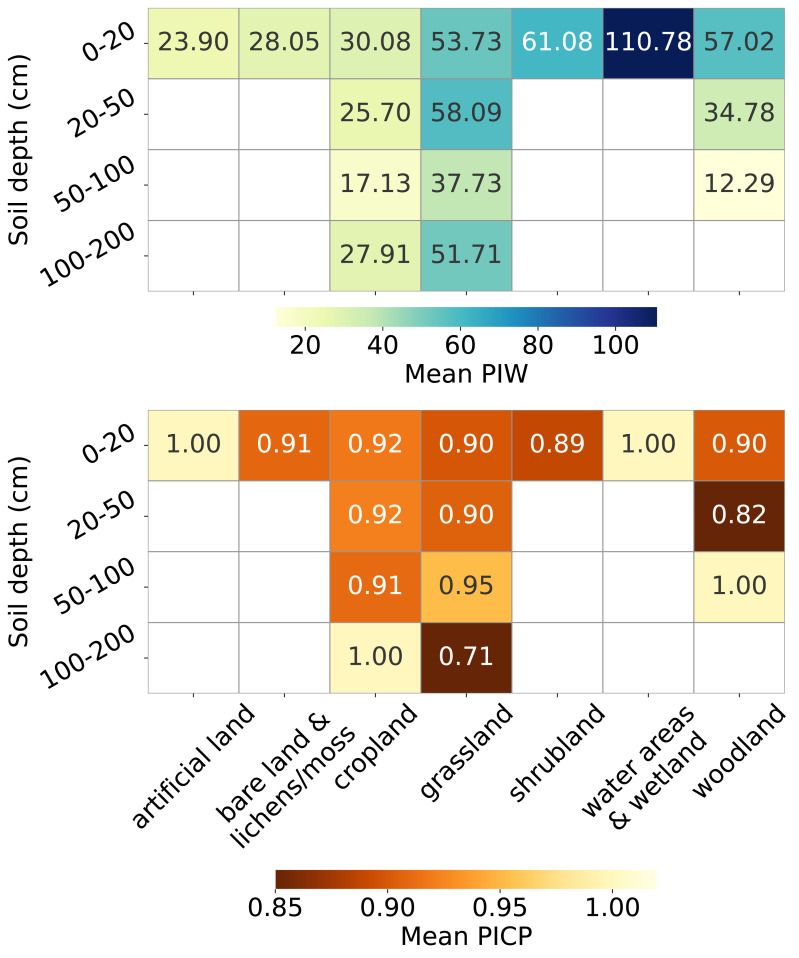
Mean PIW (left) and mean PICP (right) across combinations of land covers and soil depth intervals.

Figure 13 presents the uncertainty estimation metrics, PIW and PICP, across combinations of soil cover and depth strata. For topsoil, PIW varies significantly across land covers: it is very high for water areas and wetlands (over 100 kg/m^3^), high for woodland, shrubland and grassland (exceeding 50 kg/m^3^), relatively lower for cropland and bare land and lichens or moss (around 30 kg/m^3^), and lowest for artificial land (around 20 kg/m^3^). PIW decreases with depth for all land covers up to 100 cm.

 The PICP also varies across land covers in topsoil, with over-pessimistic estimates for artificial land and water areas and wetlands (PICP = 1), and slightly over-optimistic estimates for other land covers (PICP < 0.95). In cropland, PICP remains stable with increasing depth until the 100–200 cm interval, where it reaches 1. For grassland, PICP improves at depths of 50–100 cm (0.90 to 0.95) but drops sharply to 0.71 for depths greater than 100 cm. In woodland, PICP drops to 0.82 at depths of 20–50 cm and increases to 1 in the 50–100 cm soil depth interval.

### Maps examination

**Figure 14:**
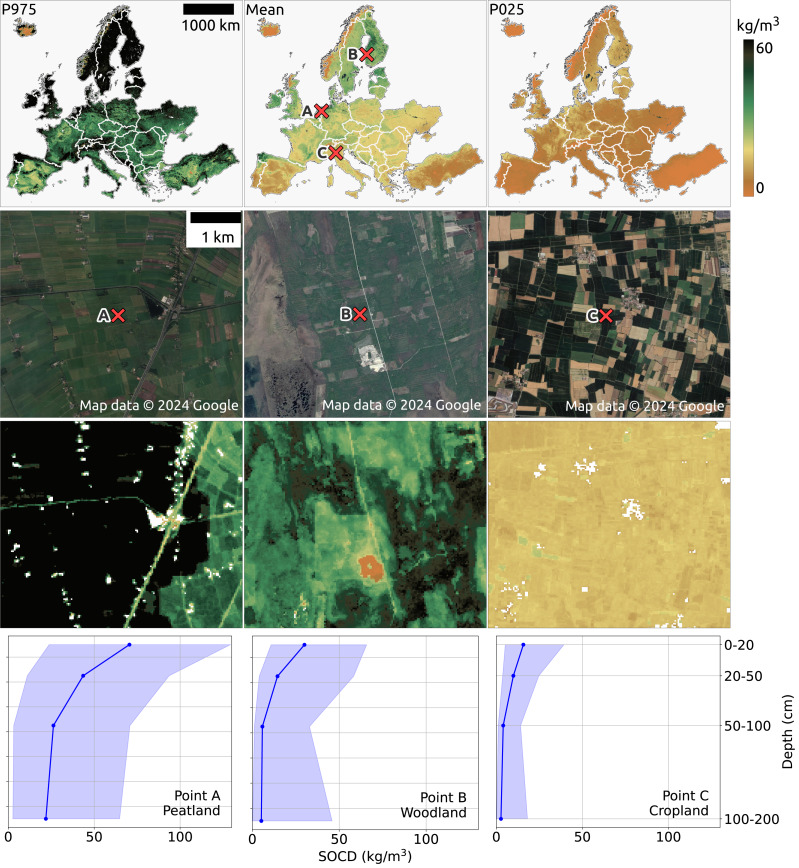
SOCD predictions for the topsoil (0–20 cm) across continental Europe from 2020 to 2022 (available at https://doi.org/10.5281/zenodo.13754343). The top row presents an overview of the predictions (middle), including the upper bound (P0.975 at left) and lower bound (P0.025 at right). The middle two rows provide zoomed-in SOCD predictions and corresponding satellite images for the same period at three specific locations: (A) a peatland site in the Netherlands (lon: 6.177, lat:52.583), (B) a woodland area in Finland (lon: 22.531, lat: 62.351), and (C) a cropland site in Italy (lon: 10.339, lat: 45.101). The bottom row illustrates the variation of SOCD predictions with depth at these three points, with the pale blue shading indicating the corresponding PI. Google Maps (2024, CNES/Airbus, Maxar Technologies), available through https://www.google.com/maps/, last accessed: 30 August 2024.

Figure 14 (top) presents the pan-European maps of SOCD predictions and the corresponding PIs for the period 2020–2022 for topsoil at a depth of 0 cm to 20 cm. In general, SOCD predictions are higher at higher latitudes and lower at lower latitudes, with the lowest values observed in Spain and Türkiye. In addition to the SOCD prediction maps, the P025 map (lower bound of PI: 2.5th percentile) and the P975 map (upper bound of PI: 97.5th percentile) are also shown in the top of Fig. 14. The P025 map exhibits minimal spatial variation, while the P975 map mirrors the spatial patterns of the SOCD predictions, showing higher values in regions with greater latitude and altitude. Consequently, PIs are wider in areas with higher SOCD predictions, consistent with observations in Fig. 12.

The second and third rows of Fig. 14 provide three zoomed-in examples of SOCD prediction maps for the period 2020–2022 at the topsoil level, centered on three specific locations: (A) a peatland site in the Netherlands, (B) a woodland area in Finland, and (C) a cropland site in Italy. The peatland site (A) exhibits the highest SOCD, while the cropland site (C) shows the lowest, with the woodland site (B) falling in between. The bottom row illustrates SOCD variation across different depth intervals for the three locations. A general trend of decreasing SOCD values with increasing depth is observed at all three sites, with spatial variation diminishing as depth increases. Although SOCD decreases with depth, it remains relatively high in deeper layers at the peatland site, while dropping to very low levels in the cropland. The PIW is smallest in the cropland and largest in the peatlands. For the peatland site, PIW remains relatively constant across depths, whereas for the woodland and cropland sites, it decreases with depth but increases again at the 100–200 cm interval.

**Figure 15:**
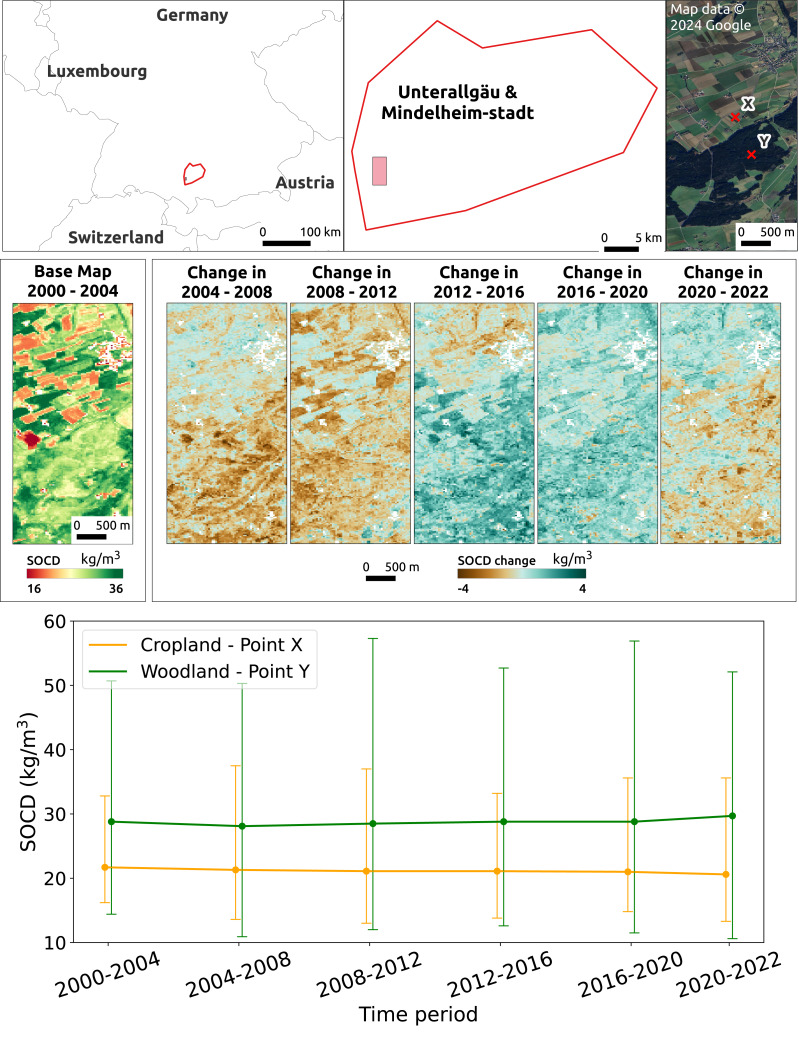
Temporal changes in SOCD at the farm scale. The top displays the location of the study area in the NUTS3 region of Unterallgäu, Bavaria, Germany, near Mindelheim Stadt, along with its landscape from satellite images. The middle presents SOCD predictions for 2000–2004 as the baseline map, followed by maps showing changes in SOCD for each subsequent 4-year interval (*e.g.*, 2004–2008, 2008–2012), calculated as the difference from the preceding interval. The middle presents SOCD predictions in 2000–2004 as a base map, and show the changes happened in the consecutive years compared to previous year intervals from 2004 to 2022. The bottom shows the time series of SOCD predictions and corresponding 95% PI for two points: X (cropland, long: 10.159, lat: 47.937) and Y (woodland, lon: 10.163, lat: 47.929). Google Maps (2024, CNES/Airbus, Maxar Technologies), available through https://www.google.com/maps/, last accessed: 30 August 2024.

Figure 15 illustrates the temporal changes in SOCD on the farm scale for a location in the NUTS3 region of Unterallgäu, Bavaria, Germany, near Mindelheim Stadt, including both cropland and woodland. To emphasize the contrast in SOCD changes, a binary color map was applied with the color range compressed between 16 kg/m^3^ and 36 kg/m^3^. The figure shows that during the period 2004 to 2012, SOCD values decreased in the forested area while remaining relatively stable in the cropland area. After 2012, SOCD values increased. Overall, the changes in SOCD are relatively small. Two points, X and Y, were selected from cropland and woodland, respectively, to illustrate the time series of SOCD predictions and corresponding PIs at the pixel level, as shown in the lower of Fig. 15. Over time, both points exhibited very small variations, especially compared to their respective PIs. The uncertainty level is notably high, with a PIW of approximately 15 kg/m^3^ for cropland and 35 kg/m^3^ for woodland.

**Figure 16:**
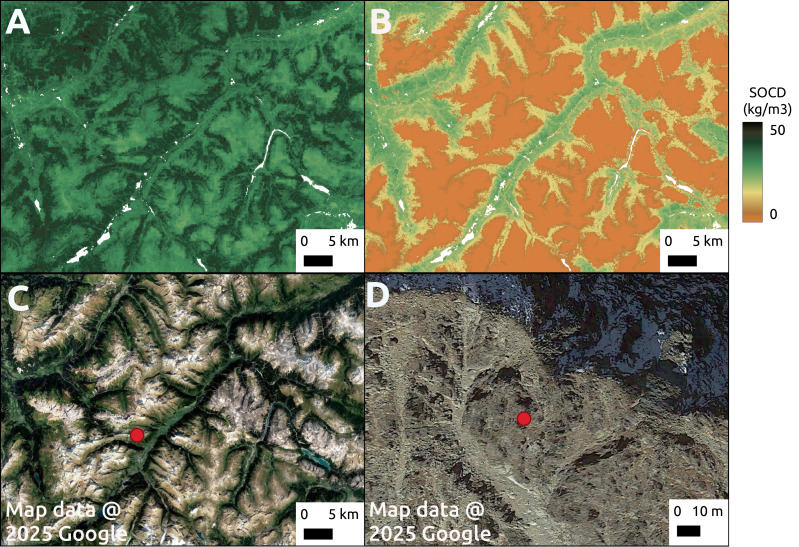
Comparison of model predictions without (A) and with (B) the inclusion of pseudo-zero points during training. The comparison is shown for a region in the Alpine mountains (C), with a zoomed-in view of a rocky mountain peak area (D). Google Maps (2024, CNES/Airbus, Maxar Technologies), available through https://www.google.com/maps/, last accessed: 16 April 2025.

Although pseudo-zero points are excluded from the calculation of performance metrics, we can still observe their impact in the map predictions (Fig. 16). In a mountainous region of the Alps, the model tends to overestimate SOCD values on rocky mountain tops when pseudo-zero points are not included.

**Figure 17:**
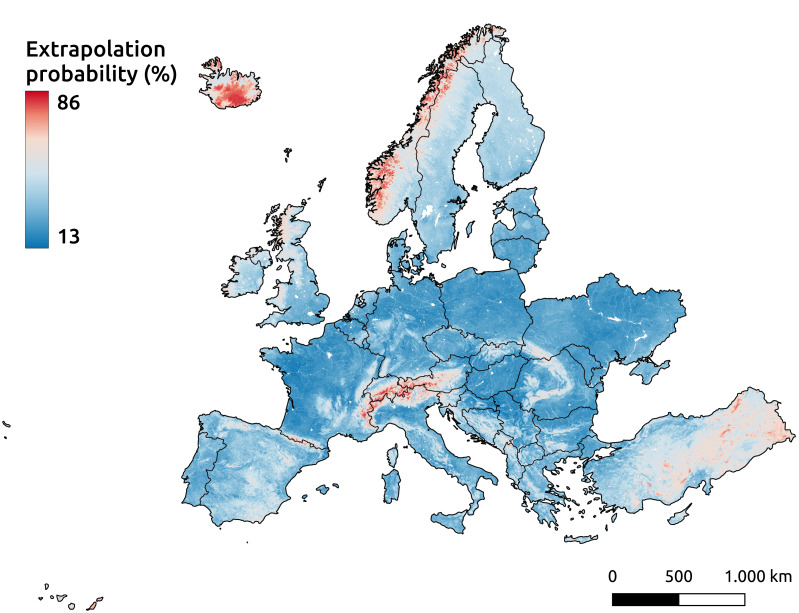
Extrapolation risk probability map for topsoil during the period 2020–2022.

Even with the inclusion of pseudo-zero points, the Alpine mountain regions remain among the areas highlighted by the extrapolation risk maps, as shown in Fig. 17. This probability map, derived using the IF, indicates higher values for areas with a greater likelihood of being unfamiliar to the model—implying a higher risk of extrapolation. The recommended threshold for determining whether an area is classified as extrapolated is 64%, represented by red in the figure. Other identified areas include the western mountainous regions of Norway, most of Iceland, and the eastern part of Türkiye.

### Spatial aggregation

**Figure 18:**
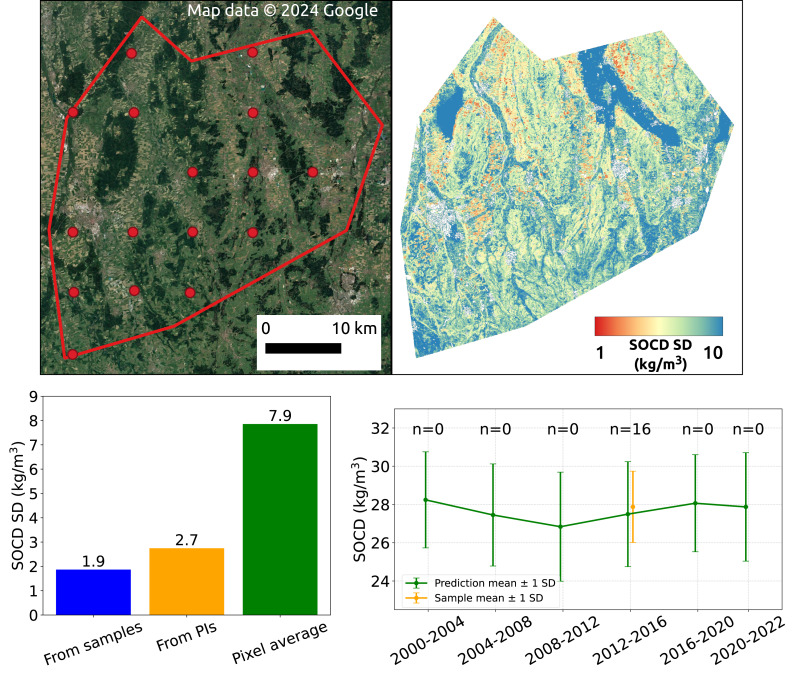
SOCD observation samples within the AOI (top left); SOCD standard deviation map of the AOI (top right); Comparison of SOCD uncertainty in AOI estimated from model-based maps, design-based samples, and the average of pixel-level uncertainty (bottom left); time series of spatial aggregates and associated uncertainty (bottom right). Google Maps (2024, CNES/Airbus, Maxar Technologies), available through https://www.google.com/maps/, last accessed: 30 August 2024.

Figure 18 illustrates the effect of spatial aggregation. The top left shows a satellite image depicting the landscape of the AOI, with red dots indicating the sample measurements available within this AOI. The top right displays the SOCD standard deviation derived from the 95% PI map. The bar plot in the bottom left compares the uncertainty estimated at the regional level from three approaches: spatial aggregation based on samples, spatial aggregation based on SOCD predictions, and the simple average of pixel-level standard deviations within the AOI for the period 2012–2016 (the only period with available samples). The plot demonstrates that spatial aggregation effectively reduces the uncertainty level from approximately 7.9 kg/m^3^ to 2.7 kg/m^3^, which is only slightly higher than the aggregated uncertainty derived from samples. The bottom right presents the time series of spatial aggregates and uncertainty for this AOI, with estimates from model predictions (mean: 27.5, standard deviation: 2.7) and design-based samples (mean: 27.9, standard deviation: 1.9), which are comparable.

## Discussion

### Key variables to explain SOCD variations

Our results show that soil_depth is the most important feature, aligning with the findings of Hengl et al. (2017) and Sothe et al. (2022). The vertical variation of SOCD and its negative correlation with soil depth have been widely reported for mineral soils (Jobbágy & Jackson, 2000; Lorenz & Lal, 2005; Gross & Harrison, 2019), and is also evident in our training data (Fig. 3), Shapley analysis (Fig. 6) and map predictions (Fig. 14). While this general trend is expected, the near-linear negative relationship observed in Fig. 6 may be influenced by the limited availability of data from deeper soil layers, which could reduce the model’s ability to capture more complex patterns at depth. An exception is also observed in the deepest layer (100–200 cm) for cropland and grassland in the training data, where SOCD increases slightly—likely also a result of data scarcity introducing bias in those specific strata (Fig. 3). This high feature importance likely reflects soil_depth’s role as the only variable distinguishing samples from different soil horizon layers at the same location and year, due to the absence of depth-specific predictor layers. Most available covariates to date primarily represent surface environmental processes, such as vegetation and climate. While Helfenstein et al. (2024a) developed depth-specific peat soil maps for the Netherlands, such data are still lacking at the EU scale. Both data scarcity and potential for deep SOC storage emphasize the importance of including depth as a dimension in SOC mapping, which is critical to evaluate climate solutions and projecting terrestrial climate change feedbacks (Lorenz & Lal, 2005; Hicks Pries et al., 2023).

Following soil_depth, vegetation indices form the second most important feature group. The positive correlation between vegetation indices and predicted SOCD reflects the critical role of vegetation in soil formation and organic matter accumulation (Kooch, Amani & Abedi, 2022). Fraction of Absorbed Photosynthetically Active Radiation (FAPAR), which quantifies the fraction of solar radiation absorbed by plants, emphasizes vegetation activity and demonstrates reduced saturation effects compared to NDVI (Robinson et al., 2018; Myneni & Williams, 1994). BSF, on the other hand, reflects bare soil exposure over a year. While they all rank highly in feature importance, further investigation is needed to know the extent to which they provide complementary information, given their mutual correlation. Vegetation conditions are known to influence SOCD through factors such as natural *versus* managed vegetation, root distributions, and the allocation of above- and below-ground biomass (Jobbágy & Jackson, 2000; Hu et al., 2018; Ramesh et al., 2019; Schneider, Poeplau & Don, 2021). These influences are reflected in both the training dataset (Fig. 3) and the resulting maps (Fig. 14). However, the reliance on vegetation features can result in unrealistic predictions in a spatial context. As shown in Fig. 15, some vegetated cropland parcels exhibit higher SOCD values than woodland, driven by correspondingly high FAPAR and NDVI values in this region (see supplementary notebook 016_check_feature (https://github.com/AI4SoilHealth/SoilHealthDataCube/blob/main/SOCD_map/016_check_feature.ipynb)).

The relationship between climatic variables and SOCD is complex and often non-linear (Fig. 6). Numerous studies have suggested the associations between SOCD and climatic variables such as temperature, precipitation, and moisture (Jobbágy & Jackson, 2000; Yang et al., 2008; Luo et al., 2017; Gross & Harrison, 2019; Kerr & Ochsner, 2020). Beyond direct time-series data, pattern- or characteristic-based features derived from time series have shown significant value in predicting SOCD. For instance, bio3_longterm_m, which quantifies day-to-night temperature oscillations relative to seasonal changes, is the most important temperature-related feature, despite the inclusion of LST time series. The impact of temperature variation has also been observed in laboratory experiments (Adekanmbi et al., 2022), although further research is needed to understand this phenomenon on a continental scale in Europe. These highlight the importance of feature engineering in extracting meaningful patterns from raw data to improve model efficiency and accuracy in predicting SOCD. Understanding these interactions is crucial for anticipating how SOCD might respond to climate change (Crowther et al., 2016).

Other features that were selected but did not rank among the top ten included topographic features, lithology.peat_static_c, and backscatter.vv_static_m. As a key soil-forming factor, topography influences soil water status, as well as erosion and deposition processes (Jenny, 1994; Behrens et al., 2010). The feature lithology.peat_static_c, the only selected feature from the lithology group, is particularly important in areas with a high probability of being peatland (Fig. 7A). Backscatter information, represented by backscatter.vv_static_m, ranked relatively low among the selected features. Its influence on SOCD predictions may come from its relationship with soil moisture (Ceddia et al., 2017). Although not among the most important features, these variables contribute to the prediction by providing unique information not captured by other feature groups.

In addition to examining Shapley values globally and comparing them across different locations, another valuable question is how Shapley values vary for the same point across time and depth (Fig. 6). Such an analysis could potentially provide insights into prediction variability over temporal and depth dimensions. However, addressing this question would require a more thorough and dedicated research effort, which is beyond the current study’s focus. We expect further research to explore this important topic.

By quantifying the contribution of each feature to a model’s predictions, Shapley values provide insights into the model’s decision-making process. However, it is important to note that these values do not imply causality. The interpretations provided (Fig. 6) are conditional on the data and features used. The dataset does not fully represent continental Europe, as shown in Fig. 2. Additionally, while an extensive set of feature layers was selected to capture the underlying mechanisms of SOCD, these features have inherent limitations. They offer only a sampled approximation of true conditions across space and time, potentially missing critical information. Furthermore, the process of generating these features involves errors and uncertainty. Therefore, as emphasized by Wadoux, Saby & Martin (2023), these features should be regarded as proxies rather than definitive representations.

### Interpretation of overall model performance and spatial patterns

Overall, the model shows good predictive performance, with predicted values closely aligning with observations (Fig. 8). Compared to the independent test results, performance metrics under ISIW-CV show only minor declines. This suggests that, despite the clustered nature of the sampling data, its representativeness is not significantly compromised—likely due to the broad spatial coverage provided by LUCAS across the EU. While the overall performance is good, the model tends to overestimate low SOCD values and underestimate the high values, consistent with findings from previous studies (Hengl et al., 2017; Sanderman et al., 2018; Feeney et al., 2022; Sothe et al., 2022). This may partly reflect the smoothing effect typical of RF, an ensemble learning algorithm.

To improve the model’s performance in capturing low SOCD values, two approaches are adopted: (1) inclusion of pseudo-zero points, and (2) application of a log-transformation. In soil surveys, deeper layers and non-soil areas—such as sand dunes and bare rocks—are often overlooked due to practical constraints. This results in a lack of representation for “absence of soil” scenarios in the feature space, leading to model confusion at the lower end of the SOCD scale. To address this, pseudo-zero points were introduced. Although excluded from performance metric calculations—both to rely solely on real measurements and to avoid skewing variance-based metrics like R^2^ and CCC due to their clustering at the origin—they still influence the predictions (Fig. 16). Note that these pseudo-zero values are only used at the surface level. Log-transformation also helps mitigate this issue, as suggested by Hengl et al. (2017) and Guevara et al. (2020). When the model is trained on the original linear scale, the overestimation of SOCD values can also be attributed to the loss function of the RF regressor, squared error, which places greater weight on extreme values and emphasizes high-variance points, often linked to higher SOCD. Log-transformation can mitigate this but may reduce accuracy for high SOCD values (see supplementary notebook 005_transformation_comparison (https://github.com/AI4SoilHealth/SoilHealthDataCube/blob/main/SOCD_map/005_transformation_comparison.ipynb)). From a user perspective—such as for agricultural communities—accurate prediction of mineral soils is often more critical, making log-transformation a practical choice in such contexts.

When observed SOCD values exceed approximately 75 kg/m^3^, the model outputs remain below this value (Fig. 8). This is further reflected in its uncertainty estimation. As shown in Fig. 12, PIW become significantly wider for high SOCD values, while PICP exhibit instability. This is likely due to the limited availability of datasets with high SOCD training points in certain years (Fig. 2). Many legacy point datasets (*e.g.*, Batjes et al., 2017) over-represent agricultural or mineral soils, which can introduce systematic bias. This issue becomes less pronounced when more high-SOCD data are available (Sothe et al., 2022; Helfenstein et al., 2024b). Agricultural and mineral soils behave differently from organic soils, as emphasized by De Brogniez et al. (2015) and Feeney et al. (2022). While Helfenstein et al. (2024b) utilized peat occurrence across different times and depths as a feature to effectively distinguish between mineral and organic soils, such a layer is still lacking for continental Europe. Data quality may also contribute to this issue. Ziche et al. (2022) reported inconsistencies in the LUCAS 2015 dataset for forest soils, where some samples mix organic layers with mineral soils, potentially leading to underestimation. While this study uses LUCAS 2018 data instead of LUCAS 2015, further investigation is necessary to rule out similar problems.

The spatial patterns of both SOCD predictions and PI estimates generally align with the results of Poggio et al. (2021) and Van Wesemael et al. (2024). However, discrepancies are observed in regions such as part of the Alps, the northwest of Scotland, the mountainous areas of western Norway, and Iceland. These regions are characterized by bare surfaces, snow coverage and high altitudes. The discrepancies are likely attributable to the inclusion of pseudo-zero points.

The quantified PI appears to be primarily influenced by the corresponding SOCD values, with higher predicted SOCD values associated with wider PIs (Fig. 12). This relationship is also evident in Fig. 14, where the P975 map approximately mirrors the spatial variation of SOCD predictions, while the P025 map remains relatively low across most regions. In contrast, the spatial pattern of extrapolation risk probability (Fig. 17) differs and aligns more closely with the data availability distribution (Fig. 2) with highest risk of extrapolation likely happening in high altituded. Regions with limited data availability and high mountains, such as Türkiye, Norway, Iceland, and Switzerland, exhibit relatively high extrapolation risk probabilities. Interestingly, Ukraine, despite missing data, shows a low extrapolation risk probability, possibly because its feature space is similar to other well-represented regions, such as Poland or Germany. More analysis is needed to confirm this hypothesis. A simple correlation analysis quantitatively confirms the weak correlation between PIW and extrapolation risk probability (see supplementary notebook 011_aoa_isolation.tree (https://github.com/AI4SoilHealth/SoilHealthDataCube/blob/main/SOCD_map/011_aoa_isolation.tree.ipynb)), a finding that has also been reported by Hateffard, Steinbuch & Heuvelink (2024). Further investigation is required to provide an explanation, which is beyond the scope of this study.

### Model performance across land covers and soil depths

Overestimation and underestimation issues are particularly pronounced for the most under-represented land covers, water areas and wetlands and artificial land. Exhibiting the highest average SOCD values, fewest observations are available for water areas and wetlands, and the model significantly underestimates these values. Conversely, the model overestimates SOCD values for artificial land. Their extremely limited data availability make the other performance metrics less informative.

Shrubland and bare land and lichens or moss also have limited data availability, but not spare enough to exlude CCC analysis. Shrubland shows poor prediction accuracy and high model uncertainty, while bare land and lichens or moss achieve the best prediction accuracy and minimal model uncertainty among all land covers. This contrast may be explained by differences in the feature space. Shrubland is similar to other vegetated land covers, such as grassland, in terms of above-surface characteristics—despite differences in rooting systems and below-ground SOCD distribution (Jobbágy & Jackson, 2000; Lorenz & Lal, 2005). However, our feature set lacks information that captures these below-ground distinctions. In contrast, bare land and lichens or moss can be more easily distinguished due to its distinct and detectable surface features.

Cropland, grassland, and woodland are the three land covers with sufficient data at deeper soil layers. All show moderate prediction accuracy at the topsoil level (0–20 cm) and improved accuracy at the 20–50 cm soil depth interval, as reflected in narrower PIs. This improvement may result from reduced spatial variability in deeper soils—allowing limited data to better represent the underlying patterns—or it may simply reflect that the reduced data availability at these depths has already lost its informative value. Only grassland and cropland have sufficient data for analysis beyond the 50 cm soil depth. However, the variation in their prediction accuracy across depths still requires more detailed analysis and additional data to fully understand.

Compared to the model’s performance on the entire test dataset, its performance across specific land cover types and soil depth intervals is suboptimal for both prediction and uncertainty estimation. This is expected, as global model performance often declines when evaluated on stratified subsets—particularly those with limited data availability. As shown in Figs. 2 and 17, additional data from underrepresented regions, such as Norway, Switzerland, and Türkiye, are needed to fill current gaps and avoid extrapolation risk. Improved data availability for deeper soil layers, particularly for the 100–200 cm interval, is essential to reduce uncertainty at these depths. This could involve both collecting more measurements and inserting pseudo-zero values derived from horizon surveys, which are often recorded separately from SOC measurements. Furthermore, increasing the representation of high-SOCD samples would be highly beneficial.

### Reduced uncertainty at larger spatial support

In this work, model-predicted uncertainties are presented as PI maps along with SOCD predictions. We consider this necessary for several reasons:

1. it prevents misleading summary metrics by highlighting prediction uncertainty (Veronesi & Schillaci, 2019);
2. it identifies conditions where the model may fail or produce unreliable predictions, aiding responsible decision-making (Wadoux, Minasny & McBratney, 2020; Breure et al., 2022);
3. it enables more thorough model comparisons, especially when a model with reliable uncertainty estimates is preferred (Szatmári & Pásztor, 2019; Veronesi & Schillaci, 2019);
4. it allows for uncertainty propagation when the soil map is used in models (Heuvelink, 1998; Schmidinger & Heuvelink, 2023).

Although a 90% probability PI is commonly used (*e.g.*, Odgers, McBratney & Minasny (2015), Malone et al. (2017), Arrouays et al. (2020b), Schmidinger & Heuvelink (2023)), we opt for a 95% PI because it corresponds to ±2 standard deviations. This choice simplifies communication and comparison, although a correction factor may still be required if the data distribution deviates from normality (see the supplementary notebook 013_variogram_spatial.aggregate (https://github.com/AI4SoilHealth/SoilHealthDataCube/blob/main/SOCD_map/013_variogram_spatial.aggregate.ipynb)).

The uncertainty level (95% PI) is high at the pixel level, especially for pixels with high SOCD values. In some cases, the PIW even exceeds the corresponding predicted SOCD values (see Figs. 14 and 15). However, this uncertainty can be significantly reduced by spatial aggregation on larger supports, as shown in Heuvelink et al. (2020), Szatmári, Pásztor & Heuvelink (2021), Wadoux & Heuvelink (2023), Emick et al. (2023) and Szatmári et al. (2024). Larger spatial supports result in smaller uncertainties associated with aggregated predictions due to the weak spatial autocorrelation of standardized map errors. This approach is valuable for revealing temporal changes and regional comparisons using a larger support, which are crucial for policy and decision-making.

The regional averages and uncertainties derived from the model predictions are consistent with those estimated from the samples, although not necessarily more accurate. However, sample-based estimation depends on the availability of sufficient measurements, which is not always feasible (Emick et al., 2023). For example, as shown in Fig. 18, measurement samples are available only for the 2012–2016 period, limiting the formation of long-term time-series. In such scenarios, model predictions serve as a viable alternative.

This study demonstrates the method and impact of spatial aggregation using the maps presented at the NUTS3 regional level for a single region. For a more comprehensive evaluation of the impact of spatial aggregation, it would be beneficial to test different spatial supports, as done by Szatmári, Pásztor & Heuvelink (2021) and Szatmári et al. (2024). In addition, exploring the aggregation in various regional classifications, such as soil regions, represents a valuable direction for future research.

### Challenges in detecting temporal SOCD changes

In our study, static features were found to have a stronger influence on model predictions than dynamic ones, aligning with findings from previous research (Heuvelink et al., 2020; Szatmári, Pásztor & Heuvelink, 2021; Szatmári et al., 2024). This dominance is also reflected in the largely stable map predictions over time (Fig. 15). It can be partly explained by the nature of many key features—such as topography—which change very slowly over time and are typically only available in static form. Even when both static and dynamic versions of a variable were available, the model often favored the long-term (static) representations. This preference for static features may reflect the slow-changing nature of SOCD (Poeplau & Don, 2013), whose changes may be too subtle to stand out over a 20-year period. At the same time, spatial variability in SOCD across continental Europe is strong and consistent, which can overshadow subtle temporal dynamics. For example, while high NDVI values are generally associated with high SOCD across space, changes in NDVI can occur rapidly, whereas SOCD responds much more gradually and may not show immediate change. This indicates that predictions are driven primarily by spatial patterns rather than temporal variation. This suggests that while the model performs well at capturing the spatial distribution of SOCD, its ability to detect changes over time—especially subtle or short-term changes—is more limited.

Furthermore, in our spatiotemporal model, each observation—defined by a unique combination of time, location, and depth—is treated independently. This means the model does not explicitly capture temporal correlation. Instead, it can only infer temporal patterns from the values of dynamic predictors. This may further limit its sensitivity to temporal dynamics and cause it to over-focus on patterns learned from broad spatial scales. Temporal correlation includes not only the autocorrelation of SOCD itself but also its relationships with the time series of environmental predictors. Some studies have attempted to incorporate such relationships in DSM to distinguish subtle temporal changes from dominant spatial patterns and to account for the delayed response of SOCD to environmental drivers. For example, Heuvelink et al. (2020) introduced a decayed NDVI feature to account for a 36-year lag, while Helfenstein et al. (2024b) used historical land-use maps to incorporate the most frequently occurring land-use types from years prior to the prediction point in the Netherlands. However, such approaches remain rare, largely due to the limited availability of repeated sample measurements, the intensive effort needed to compile long-term datasets, and the lack of established methods for integrating temporal correlation into model structures.

Among the dynamic features—which introduce the temporal dimension into the models and resulting maps—climate-related variables generally showed higher importance than vegetation-related ones. While climate parameters are traditionally defined as long-term summaries of weather conditions, in this context, “climate-related features” also include higher-resolution time-series, such as monthly precipitation and LST, which ranked higher than vegetation indices. This pattern may reflect two factors: first, the underlying environmental dynamics, as variables like precipitation and LST tend to fluctuate more over time than vegetation cover; and second, the higher temporal resolution at which climate-related data are typically available, offering richer input for the model. Notably, precipitation was the most important among all dynamic features, possibly due to its more direct influence on soil properties. Another factor is that climate features often lack consistent long-term counterparts, while vegetation indices typically exist at multiple temporal resolutions. These observations also highlight the need for caution when interpreting feature importance. Although it can offer insights into model behavior and potential environmental processes, feature importance is also shaped by the way input layers are structured and the quality of the underlying data. As such, it should not be overinterpreted as a direct reflection of ecological or pedological processes, but rather understood as a product of both environmental relevance and data representation.

The model’s temporal transferability was assessed using LOYO-CV, which resulted in lower performance metrics compared to the independent test set. This decline is likely due to the exclusion of specific datasets when individual years are omitted from the training data. For instance, the LUCAS SOCD dataset, which is only available for 2018, is entirely excluded when this year is left out, resulting in more spatially clustered training data. Consequently, the evaluation of temporal transferability is partly influenced by the challenges associated with spatial clustering. The challenge of evaluating temporal transferability is further illustrated in Fig. 9. The figure shows that low training data availability does not consistently lead to poor performance. Instead, performance appears to be more influenced by the proportion of data points across cropland and non-cropland areas. Since the temporal dimension is introduced primarily through dynamic features, we argue that model performance depends more on representativeness in the feature space than on the temporal coverage itself.

Consistent, repeated soil sampling is critical for assessing model performance in temporal dimensions, analyzing temporal correlations in SOCD and enabling their integration into modeling approaches. While Helfenstein et al. (2024b) assessed model’s accuracy across time using datasets spanning 1952 to 2018 in the Netherlands, extending such analyses to larger spatial extents remains challenging due to the lack of such validation data with repeated SOCD measurements. Unlike established networks such as Fluxnet for flux measurements (https://www.europe-fluxdata.eu/home/sites-list) or the International Soil Moisture Network (https://ismn.earth/en/), there is currently no extensive long-term monitoring network for soil, apart from LUCAS. To date, only three rounds of repeated SOC measurements are available. Thus, in this study, the temporal model uncertainty and the ability to predict SOCD changes were mainly analyzed through estimated PIs, assuming that model predictions are valid and their uncertainty is effectively captured by the PIs, which serve as a reference to identify potential SOCD changes. As discussed in the previous section, pixel-level PIs can be quite high, making it challenging to reliably identify areas with statistically significant SOCD changes. Aggregating predictions over larger spatial supports reduces prediction uncertainty, facilitating the identification of statistically significant SOCD changes over larger spatial extent (Szatmári, Pásztor & Heuvelink, 2021; Szatmári et al., 2024).

In summary, given the current time frame of 2000–2022, the lack of repeated samples, the absence of temporal correlation in the model, and the predominance of spatial patterns over temporal ones in the model, drawing reliable conclusions about SOCD changes from model predictions remains challenging. As highlighted by Broeg et al. (2024) and Helfenstein et al. (2024b), even though the spatio-temporal model demonstrates good general performance, this does not necessarily ensure comparable temporal accuracy in predicting SOCD changes or trends. Moreover, the estimated uncertainty of SOCD predictions cannot be directly translated into the uncertainty of SOCD changes, as this requires accounting for cross- and spatial correlations in prediction errors (Helfenstein et al., 2024b). Consequently, using such a model for temporal monitoring or detecting SOCD change should be approached with caution. Although PIs can help assess the reliability of predicted SOCD changes, areas where changes exceed the PIs—or where consistent PI patterns align with the direction of change—should be interpreted as indicators of potential change, not as confirmation that real-world change has occurred.

### Future prospects

This mapping framework provides a baseline time-series of spatially continuous, high-resolution SOCD maps for continental Europe. However, as briefly covered in previous sections, several potential improvements have been identified, some of which are already included in our implementation plan:

• **Include more representative soil data to fill spatial and temporal gaps:** More frequent sampling over time, along with additional data to improve representativeness in both spatial coverage and feature space, is essential for increasing the model’s generalization ability (Yuzugullu et al., 2024). Continued support for, and expansion of, initiatives like LUCAS is critical to establishing a consistent, long-term monitoring framework capable of providing such data. The forthcoming LUCAS 2022 dataset, providing a fourth round across the EU, marks an important step in this direction.
• **Pay equally attention to bulk density and coarse fragment data:** While SOC content data are relatively abundant, corresponding bulk density and coarse fragment data are often overlooked, , limiting the ability to accurately quantify SOCD—especially in the context of climate change and carbon farming (Poeplau, Vos & Don, 2017).
• **Introduce more relevant, appropriately developed features:** Addressing process gaps requires the inclusion of representative features—such as those capturing deeper soil conditions, distinguishing organic soils, and introducing temporal correlations.
• **Develop a map for depth to bedrock:** This would enable more complete estimation of SOCD across the full soil profile and help users interpret SOCD maps at different depths. However, achieving this will require significant effort and data integration.
• **Explore of new model structures and approaches**: Experiments with alternative model approaches or structures, such as training separate models (De Brogniez et al., 2015; Van Wesemael et al., 2024) or hierarchical model (Nussbaum et al., 2023) for each soil type and subsequently assessing their accuracy, could potentially improve model performance.
• **Maintain and update the maps:** The maps are planned to be updated regularly using a *‘re-analysis’* approach, as is common in climate science (*e.g.*, Dee et al. 2014). We recommend that users base their analyses on the latest version of the data. Although large-scale changes between versions are unlikely, regional updates may occur where new data begin to fill gaps, potentially revealing new insights. This update could also be challenging, as many feature layers used in mapping are no longer updated. For example, CHELSA climatic features, which rank high in importance, is no longer updated beyond 2018 (https://chelsa-climate.org/timeseries/). Suitable alternatives need to be identified.
• **Optimize computational efficiency:** Deriving PIs and extrapolation risk probability is essential for ensuring map reliability but significantly increases computational costs, particularly for finer spatial or temporal resolutions. To minimize compromises in map accuracy, efforts should be directed toward improving computational efficiency.
• **Seek for ways to validate block predictions:** Validating block predictions over multi-year periods and depth intervals is challenging. Direct sampling for such validation is infeasible, so legacy data and CV within the spacetime block of interest must be leveraged. Distinguishing between point predictions (*e.g.*, for 2022 at a depth of 10 cm) and block predictions (*e.g.*, for 2020–2024 at a depth interval of 0–20 cm) is critical. Validation of block predictions would require composite samples over multi-year periods, which may not always be feasible.
• **Improve the communication of map uncertainties to the users**: For specific locations or use cases, the integration of SOCD maps, uncertainty maps, extrapolation risk maps, and local knowledge is essential for mitigating potential risks associated with the use of this map product and similar tools in soil carbon farming, as well as for addressing credibility concerns (Moinet et al., 2023; Paul et al., 2023). Efforts have been made to provide users with as thorough accuracy-related information as possible. While we emphasize the importance of transparency and thoroughness regarding model and map accuracies, we also recognize that this level of detail may pose challenges for users (Padarian & McBratney, 2023). Therefore, there is a need to develop improved methods for effectively communicating this information to users.

## Conclusions

We developed a comprehensive framework for automated predictive soil mapping using RF in a spatiotemporal context, utilizing EO data and a harmonized SOCD dataset as its foundation. This framework maps soil carbon dynamics over an extended period (2000–2022+) at a fine spatial resolution (30 m). Quantitative evaluation demonstrated good overall prediction accuracy, though model performance declined for specific land covers and depth intervals. Validation of PI estimates confirmed that the models effectively capture uncertainty, despite the reduced accuracy for higher soil carbon density values. Exploratory analysis using Shapley values identifies soil depth as the most important feature, followed by vegetation (Landsat bimonthly biophysical indices), long-term bioclimatic variables, and topographic features. While pixel-level PIs are notably wide, spatial aggregation reduces uncertainty significantly, enabling the detection of SOCD changes at larger spatial supports. However, given the current time frame of 2000–2022, the absence of consistent, repeated samples, and the predominance of spatial patterns over temporal ones in the model, drawing reliable conclusions about SOCD changes is still challenging.

Potential applications of the data include: (1) providing a resource for spatiotemporal analysis of land degradation and restoration hotspots, and supporting soil health assessments with caution regarding its limitations; (2) offering a basis for optimizing sampling designs by considering prediction uncertainty; and (3) exploring future soil carbon potential through model extrapolation under different land use or climate scenarios. We aimed to keep the SOCD maps as complete as possible to maximize their usability. However, map performance varies. We recommend that users interpret the maps with caution, considering local knowledge, specific use-case requirements, and the accompanying uncertainty and extrapolation risk layers. Based on accuracy metrics, the maps are most reliable for topsoil applications in cropland, grassland, and woodland. They can still offer valuable insights under other conditions, but should be applied with awareness of their limitations. We plan to update predictions regularly with new data, and also ensure it’s up to date. This work was made possible by the LUCAS soil initiative (Orgiazzi et al., 2018) and contributions from several European countries. Continued collaboration among European Commission bodies, national soil agencies, and research institutes is essential to advancing open, high-quality soil data across Europe through the sharing of point data under appropriate agreements.

## Additional Information and Declarations

### Data Availability

The following information was supplied regarding data availability: The data are available at Zenodo: - Tian, X., Simoes, R., Isik, M. S., Minarik, R., Ho, Y., & Hengl, T. (2024). Spatiotemporal prediction of soil organic carbon density (SOCD) for pan-Europe (2000-2022) in 3D+T (Version v20241216) [Data set]. Zenodo. https://doi.org/10.5281/zenodo.14554501. The data are available at GitHub and Zenodo:
https://github.com/AI4SoilHealth/SoilHealthDataCube. Xuemeng Tian, Tomislav Hengl, yu-feng-ho, & d-consoli. (2024). AI4SoilHealth/ SoilHealthDataCube: v20241204-1 (v20241204-1). Zenodo. https://doi.org/10.5281/zenodo.14334987.
